# The multiformity of antennal chaetae in *Troglopedetes* Joseph, 1872 (Collembola, Paronellidae, Troglopedetinae), with descriptions of two new species from Thailand

**DOI:** 10.3897/zookeys.987.54234

**Published:** 2020-11-06

**Authors:** Sopark Jantarit, Katthaleeya Surakhamhaeng, Louis Deharveng

**Affiliations:** 1 Excellence Center for Biodiversity of Peninsular Thailand, Faculty of Science, Prince of Songkla University, Hat Yai, Songkhla, 90110, Thailand; 2 Division of Biological Science, Faculty of Science, Prince of Songkla University, Hat Yai, Songkhla, 90110, Thailand; 3 Institut de Systématique, Évolution, Biodiversité (ISYEB) – UMR 7205 CNRS, MNHN, UPMC, EPHE, Museum national d’Histoire naturelle, Sorbonne Université, 45 rue Buffon, CP50, F-75005 Paris, France

**Keywords:** Antennae, chaetotaxy, Entomobryoidea, Isthmus of Kra, phaneres, S-chaetae, subterranean habitat

## Abstract

Two new species of the genus *Troglopedetes* Joseph, 1872 (*T. meridionalis***sp. nov.** and *T. kae***sp. nov.**) are described from caves of the Thai peninsula. This is the first report of the genus south of the Kra Isthmus. The two new species have two rows of dental spines shared by all Thai *Troglopedetes*. They differ from other members of the genus mainly in the arrangement of dorsal chaetotaxy on head. The antennal chaetotaxy of the two species is analysed in detail in the second part of the paper. All types of antennal chaetae of both new species and their distribution patterns are described for each antennal segment: scales, ordinary chaetae, S-chaetae and subapical organite of Ant. IV. Twenty different types of chaetae are recognised and all except one are present in both species. The total numbers of ordinary chaetae and S-chaetae and their patterns of distribution on antenna are very similar between the two species (483 vs. 518 ordinary chaetae; 207 vs. 208 S-chaetae). Each type of chaetae has its own distribution pattern, markedly contrasted between dorsal and ventral side of antennae, and between antennal segments. This diversity of morphologies and distribution patterns and their similarity between the two species, as well as differences with other species of the same family, suggest that antennal chaetotaxy could provide powerful new characters for the taxonomy of *Troglopedetes* and related genera.

## Introduction

The genus *Troglopedetes* Joseph, 1872 is present in both edaphic and subterranean environments (Deharveng 1987) in three regions of the world: the Mediterranean basin, Africa and tropical continental Asia. The genus includes 31 species (Bellinger et al. 1996–2020), of which 12 are present and described from Thailand, all from caves of the northern and western part of the country (Deharveng 1988a, 1990; Deharveng and Gers 1993; Jantarit et al. 2016). Thailand is today the richest country of the world for *Troglopedetes*, which is diversified both in caves and in soils, with many undescribed species (Jantarit et al. 2016). The genus was only known from localities north of the Kra Isthmus, and replaced further south by the closely related genus *Cyphoderopsis* Carpenter, 1917, with most of its species still undescribed (Deharveng and Gers 1993; Jantarit et al. 2013, 2016).

The genus *Troglopedetes* was erected in 1872 by Joseph for *T. albus* Joseph, 1872 from caves in Slovenia. Later, Absolon (1907) also used the genus name *Troglopedetes* for his type *T. pallidus* Absolon, 1907 from a cave in Slovenia, without acknowledging Joseph’s publication. This is currently accepted today, although in fact *T. albus* is not a *nomen nudum*, “however meager the description may be” according to Ellis and Bellinger (1973), who then placed *Troglopedetes* Absolon, 1907 as a junior homonym of *Troglopedetes* Joseph, 1872. Hence, *Troglopedetes* Joseph, 1872 is not a *nomen oblitum* and should be considered as the valid name for the genus in order of precedence.

The genus was subsequently recorded from Thailand by Deharveng (1988a) with *T. fredstonei*, a species from Chiang Dao cave in northern Thailand, and eleven species were later described from northern and western Thailand (Deharveng 1990; Deharveng and Gers 1993; Jantarit et al. 2016). The diagnostic characters for discriminating the different Thai species were primarily based on the dorsal chaetotaxy of head and on a set of adaptive (pigmentation, eye number, antenna length, claw complex) and non-adaptive (spines on dens) characters. Interspecific differences also exist for the length of chaetae of the labial basis and macrochaetotaxy.

*Troglopedetes* is one of the best-defined genera of Paronellidae by the subdivision of its 4^th^ antennal segment, a feature unique in the family. However, other important taxonomic characters have not been described in detail in many species, like the dorsal chaetotaxy of head and body, S-chaetal pattern on antennae and tergites, trichobothria complex, arrangement of pseudopores, and furcal structure. Regarding the antennae, like for other Entomobryoidea, only a few chaetae have been considered in published descriptions, mostly those of the sensorial organ of the third antennal segment. The complexity of antennal chaetotaxy in Entomobryoidea, with its large number of chaetal types and strong polychaetosis, may explain why authors have been so reluctant to analyse these organs in great detail. Recent works, however, by Fanciulli et al. (2003) and Soto-Adames et al. (2014) depicted more details of antennal chaetal patterns, focusing on a group of subcylindrical S-chaetae on antennal segments II and III of European species. Also, attempts to categorise antennal phaneres by Lukić et al. (2015, 2018) for Heteromurus (Verhoeffiella) and Jantarit and Sangsiri (2020) for *Alloscopus* point to the potential interest of more thoroughly investigating the antennal chaetotaxy of *Troglopedetes*.

In this study, we describe two new species, *T. meridionalis* sp. nov. and *T. kae* sp. nov., from caves of peninsular Thailand. These records are the first report of the genus south of the Kra Isthmus, considered so far as a probable biogeographical transition zone between the genus *Troglopedetes* and the closely related genus *Cyphoderopsis* (Deharveng and Gers 1993; Jantarit et al. 2013). In a second part of the paper, we describe in detail the morphological diversity of antennal chaetae of the two species as well as their arrangements. The objective is to provide a reference framework for homologising as far as possible chaetae and chaetal patterns across different genera of Entomobryoidea, and for evaluating the importance of antennal chaetotaxy for the taxonomy of Entomobryoidea.

## Materials and methods

**Figure 1.**
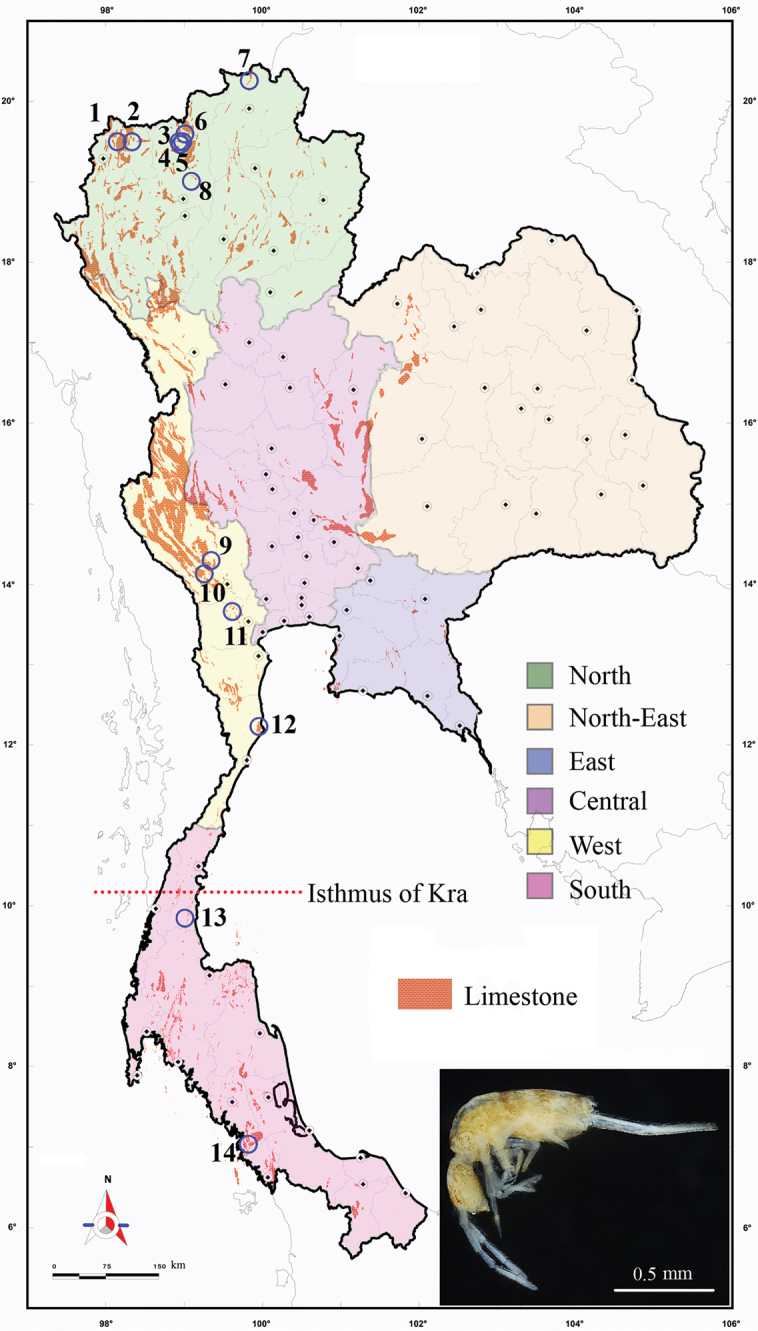
Distribution of *Troglopedetes* in Thailand (empty blue circles) with distributed limestone (orange colour) of the country **1***T. maffrei* Deharveng & Gers, 1993 **2***T. longicornis* Deharveng & Gers, 1993 **3***T. centralis* Deharveng & Gers, 1993 **4***T. fredstonei* Deharveng, 1988 **5***T. leclerci* Deharveng, 1990 **6***T. microps* Deharveng & Gers, 1993 **7***T. multispinosus* Deharveng & Gers, 1993 **8***T. maungonensis* Deharveng & Gers, 1993 **9***T. calvus* Deharveng & Gers, 1993 **10***T. dispersus* Deharveng & Gers, 1993 **11***T. convergens* Deharveng & Gers, 1993 **12***T. paucisetosus* Deharveng & Gers, 1993 **13***T. meridionalis* sp. nov. **14***T. kae* sp. nov. and its habitus (small colour picture). Small dark dots indicate province capitals; scale 1:250,000.

The specimens of the two *Troglopedetes* species described here were found in the dark zone of two caves, Tham Don Non, Lang Suan district, Chumphon Province and Tham Kae, La-ngu district, Satun Province (Fig. 1). Collembola were collected with aspirators and Berlese extractors. They were stored in 95% ethanol and were mounted on slides in Marc Andre II medium after clearing in lactic acid. Morphological characters were examined using Leica DMLB and Leica DM1000 LED microscope with phase-contrast. Photos of the habitus were taken by a Canon EOS 6D with Canon EF 100mm f/2.8 Macro lens and optimised by Helicon Remote software. Stacking was performed under Helicon Focus 6. Drawings were made using a drawing tube, and figures were improved with Photoshop and Illustrator CC/PC (Adobe Inc.).

**Abbreviations**

**Ant.** antennal segment

**Abd.** abdominal segment

**AIIIO** apical organ of Ant. III

**Th.** thoracic segment

**Tita** tibiotarsus

**mac** macrochaetae

**mes** mesochaeta(e)

**mic** microchaeta(e)

**psp** pseudopore

**tric** trichobothria

**ms** S-microchaeta(e)

**s** or **sens** S-chaeta(e)

**VT** ventral tube

**NHM-PSU** Princess Maha Chakri Sirindhorn Natural History Museum, Prince of Songkla University, Songkhla, Thailand

**MNHN**Muséum national d’Histoire naturelle, Paris, France.

### Conventions for describing chaetotaxic and pseudopore patterns

Pseudopore arrangement follows Deharveng et al. (2018). The formula of tergite pseudopores is given by half-tergite from Th. II to Abd. IV (Jantarit et al. 2013). The formula for labium basis chaetae follows the system of Gisin (1967) with the upper-case letter used for ciliated and lower-case letter for smooth chaetae. Labial chaetotaxy follows Fjellberg (1999). Dorsal chaetotaxy and chaetal areas of head follow Deharveng and Gers (1993), Jordana and Baquero (2005), and Mitra (1993). Dorsal macrochaetotaxy description combines notation of individual chaetae derived from Szeptycki (1979) with chaetal group notation (rationale explained below). Formula for dorsal macrochaetae and trichobothria are given by half-tergite from head to Abd. IV; for S-chaetae by half-tergite from head to Abd. V. Homologising ordinary chaetae between different taxa is not an easy task within Entomobryoidea where chaetotaxy has been strongly modified compared to the simple chaetotaxy of Poduromorpha, due to oligochaetosis, polychaetosis, variability, and usually unequally sized tergites (Yoshii 1989, Soto-Adames et al. 2014) as well as the important shift and secondary grouping of chaetae. It has been stressed for many years that homologies should be rooted in the analysis of first instars (Deharveng 1979; Szeptycki 1979; Zhang et al. 2011; Soto-Adames and Bellini 2015), but this has been done for a very few species only. Mitra (1973) recorded the development of tergite chaetae in the different stages of a species of *Callyntrura*; for Troglopedetinae, the only information we have in this respect is the description of the first instar of *Campylothorax sabanus* (Wray) by Soto-Adames (2016). For *Troglopedetes*, which is clearly oligochaetotic with several displaced mac, we opted for a cautious approach, using homologies by chaetal groups, less precise but more robust than chaeta-to-chaeta homologies, in cases of uncertainty. S-chaetae terminology of tergites follows Zhang and Deharveng (2014). The S-microchaeta (ms) of Th. II corresponds to S-chaetae type 1 of Jantarit et al. (2013) and microsensillum of Soto-Adames et al. (2014). The S-chaeta “sens” corresponds to S-chaetae type 2 of Jantarit et al. (2013) and to lateral sensillum of Soto-Adames et al. (2014).

## Taxonomy

### Family Paronellidae

#### Subfamily Troglopedetinae

In the concept of Soto-Adames et al. (2014), the tribe Troglopedetini is considered a synonym of Paronellini. This is plausible, but not supported by the last detailed redescription of *Paronella fusca* Schött, 1893, type species of the genus *Paronella*, by Mitra (1992), who gives 2+2 trichobothria on Abd. IV (vs. 3+3 in Troglopedetini), the main character separating the two tribes. Re-examination of the type material of the species is needed before accepting such a taxonomic decision.

##### Genus Troglopedetes

Taxon classificationAnimaliaEntomobryomorphaParonellidae

Joseph, 1872

0627F221-7107-5480-88C9-42134750675E

###### Type species.

*Troglopedetes albus* Joseph, 1872

Medium sized Paronellidae. Body colour white, sometimes with light orange to red pigment dots. Eyes per side 0–3. Scales present on antenna, head, body and ventral side of furca, absent on ventral tube and legs. Pseudopores on tergites arranged as 1, 1/1, 1, 1, 1 by half tergite from Th. II to Abd. IV, with a row of 4+4 pseudopores behind the posterior row of chaetae of Abd. IV. Labial basis chaetotaxy as M1M2R(r)E(e)L1(l1)L2(l2). Presence of one or two sublobal hairs on maxillary outer lobe. Antennae of various length, composed of four segments, with Ant. IV always divided into two equal or subequal sub-segments and devoid of apical bulb. Suture zone of head when visible follows divergent lines. Trichobothrial pattern 0, 0/0, 2, 3, 3. Dorsal macrochaetotaxy oligochaetotic, polychaetotic on the collar. Macrochaetae on head present as 0–7+0–7 in area dorsalis (central mac). Th. II with a compact group of 6+6 mac accompanied by 0–4+0–4 mac anteriorly. Th. III with a group of 3+3 mac accompanied by 0–1+0–1 mac anterior-externally. Claw with 0–2 inner teeth, a pair of lateral ones and a dorsal tooth. Dens elongated with one or two rows of spines, those on external row larger, more serrated than those on internal row. Mucro of various length, 2–16 × shorter than dens, with 3–5(6) main teeth, proximal tooth sometimes with 1–5 basal toothlets.

###### Remarks.

The number and arrangement of trichobothria on Abd. IV allows clear separation of Troglopedetinae (3+3 trichobothria) from other Paronellidae (2+2 trichobothria). Many characters are unknown for several species of *Troglopedetes*, including its type species. Two characters complementary to those listed by Soto-Adames et al. (2014) are discussed below. A new set of characters present in *Troglopedetes* are presented below as a result of this work.

###### Pseudopores.

Pseudopores are arranged on tergites as in other Entomobryoidea: 1, 1/1, 1, 1, 1 from Th. II to Abd. IV. Additionally, a row of 4+4 pseudopores is constant behind the posterior row of chaetae of Abd. IV (Deharveng, 1988a). These pseudopores are present in *Cyphoderopsis* (Jantarit et al. 2013) and were reported in various number (4–10+4–10) under the name “lenticular organs” *sensu*Christiansen and Bellinger (1996) in *Trogolaphysa* (Soto-Adames and Taylor 2013) and in T*roglobius* (Cipola et al. 2016). They have also been detected in Cyphoderidae (Jantarit et al. 2014), but not in other families of Entomobryoidea, pointing to relatedness between the cited genera and Cyphoderidae, which have led some Collembologists to consider Cyphoderidae as a subfamily of Paronellidae.

###### Macrochaetotaxy.

Dorsal clothing of macrochaetae is clearly oligochaetotic in the *Troglopedetes* species where it has been described. Pattern on Th. II includes a compact group of six chaetae (P3 complex of Soto-Adames et al. 2014) which is very obvious and constant. The same group, including 3–6 mac, is present in *Trogolaphysa* (Soto-Adames 2015, Soto-Adames and Taylor 2013), and a group of three or four mac in a line is found in *Cyphoderopsis* (Jantarit et al. 2013). The pattern on Th. III exhibits a group of three mac in *Troglopedetes* whereas *Trogolaphysa* shows 0–3 mac and *Cyphoderopsis* has none.

###### Dental spines.

Dens of *Troglopedetes* is elongated with either one or two rows of spines, those of the external row larger and more serrated than those of the internal one that are rather short and smooth. Soto-Adames et al. (2014) mistakenly stated that *Troglopedetes* has a single row of dental spines. In fact, species of the Mediterranean region, Africa, south-west and central Asia known so far have only one row of 8–45 dental spines, but all Thai species have two rows of dental spines (internal row with 9–45 spines, external one with fewer spines).

##### Troglopedetes kae sp. nov.

Taxon classificationAnimaliaEntomobryomorphaParonellidae

C41EAAD5-4C3D-53BE-8E86-152B09A1A080

http://zoobank.org/99807B93-C5B7-4F80-8499-3FF951526C20

Figures 2 , 3 , 4 , 5 , 6 , 7 , 8 , 9 , 10

###### Type material.

Holotype male and seven paratypes on slides (one male, four females and two subadults). Thailand: Satun Province: La-Ngu district, Tham Kae, 6°53'41"N, 99°46'44"E, 24 m a.s.l., 25 Jul 2017, S. Jantarit, A. Nilsai and K. Surakhamhaeng leg., dark zone of cave, by aspirator (sample # THA_SJ_STN04). Holotype and five paratypes deposited in NHM-PSU, two paratypes deposited in MNHN. Measurements of holotype in Table 1.

**Table 1.** *Troglopedetes kae* sp. nov., measurements in µm (from holotype).

**Head**		**Body**		**Appendages**	
Ant. I	90	Th. II	137	Man	305
Ant. II	215	Th. III	133	Dens	283
Ant. III	148	Abd. I	101	Mucro	30
Ant. IVa	136	Abd. II	130	Furca	618
Ant. IVb	138	Abd. III	144	Claw I	37
Ant.	727	Abd. IV	515	Claw II	35
Head	286	Abd. V	75	Claw III	36
		Abd. VI	71		
		Body	1,592		

###### Description.

***Habitus*.** Slightly troglomorphic, slender, with elongate legs, furca and antennae. Body length 1.3–1.6 mm. Fourth abdominal segment 3.5–6 × (n = 9, all adults) as long as the third one along dorsal axis. Furca well developed, ca. 1.6–2.2 (n = 8) × shorter than body length. Body colour white with spots of orange pigment. Eyes absent, no ocular patch.

***Chaetal types*.** Four types of chaetae on somites, appendages (except antennae) and mouthparts: scales, present on antennae I and II, head, body and furca, absent on legs and ventral tube; ordinary chaetae on all body parts; S-chaetae and trichobothria on tergites; hairs devoid of sockets on outer maxillary lobe and labial papilla. Chaetal types on antennae are much more diverse and described further separately.

**Figure 2.**
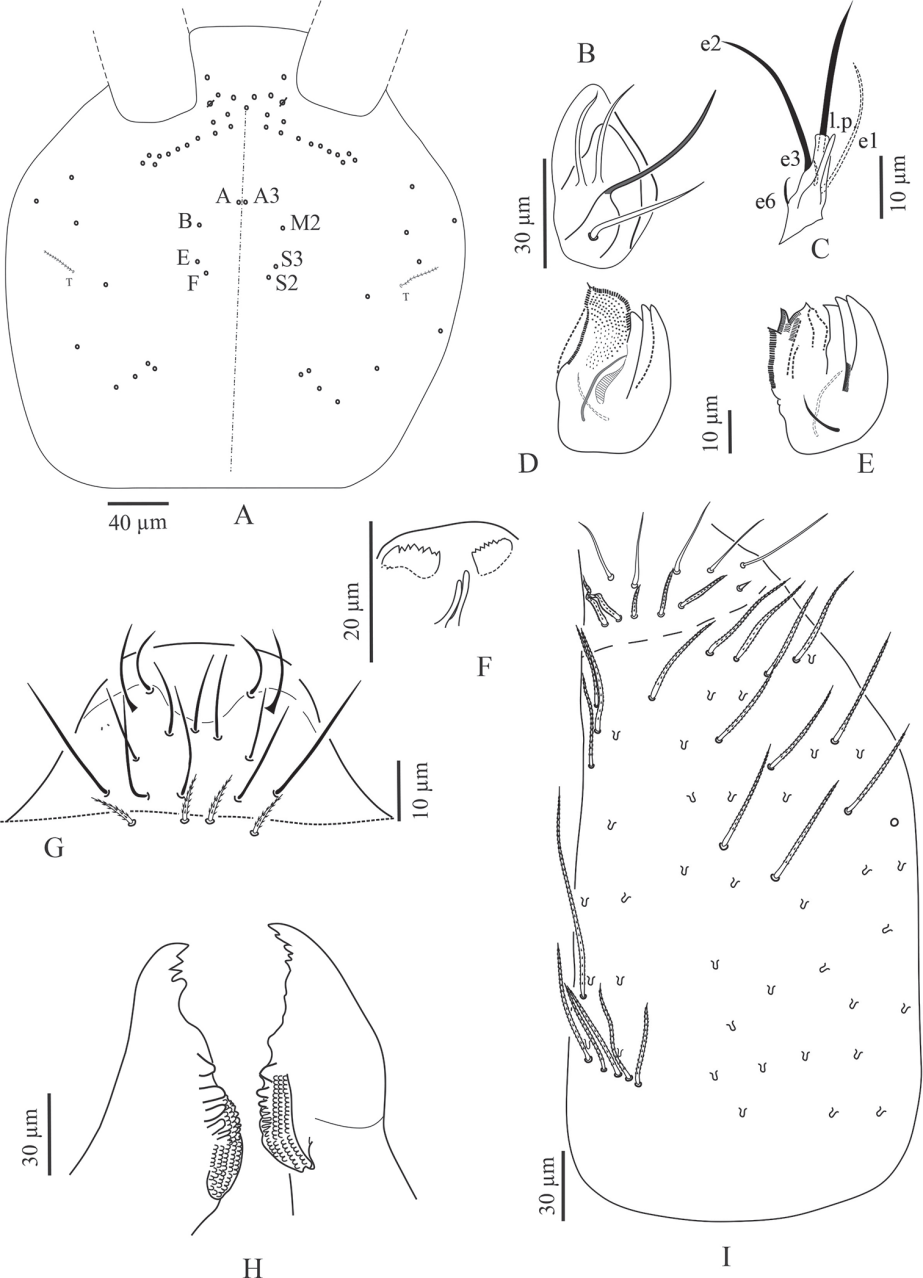
*Troglopedetes kae* sp. nov. **A** head chaetotaxy (left = **A** to **G**mac nomenclature; right = AMS nomenclature) **B** outer maxillary lobe **C** labial palp of papillae **D** maxillary head of ventral sides **E** maxillary head of dorsal sides **F** ventro-distal complex of labrum **G** labrum **H** mandible **I** labial basis and ventral chaetotaxy of head, right side.

**Figure 3.**
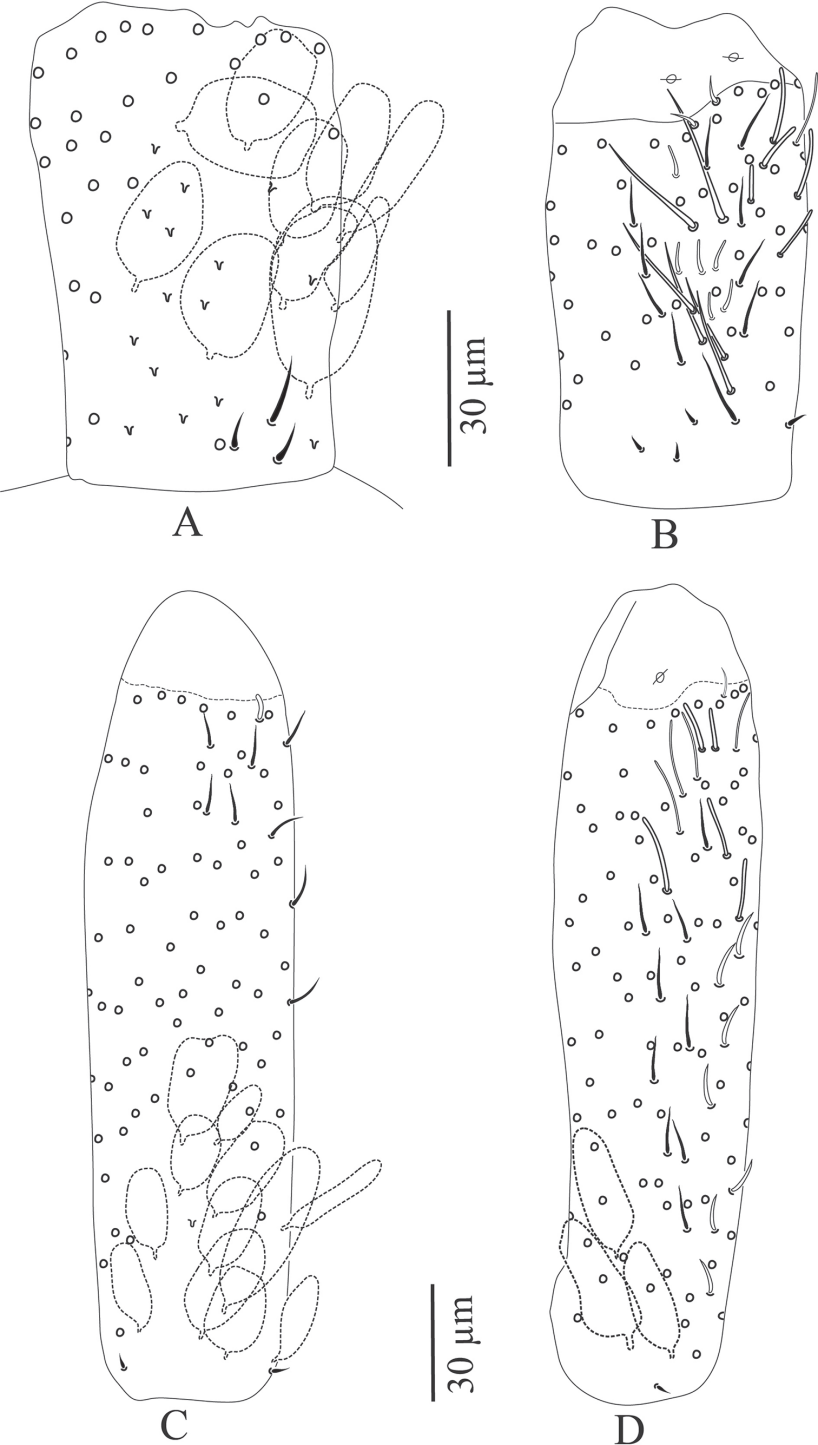
*Troglopedetes kae* sp. nov. continued **A** dorsal side of Ant. I, right side **B** ventral side of Ant. I, right side **C** dorsal side of Ant. II, right side **D** ventral side of Ant. II, right side.

**Figure 4.**
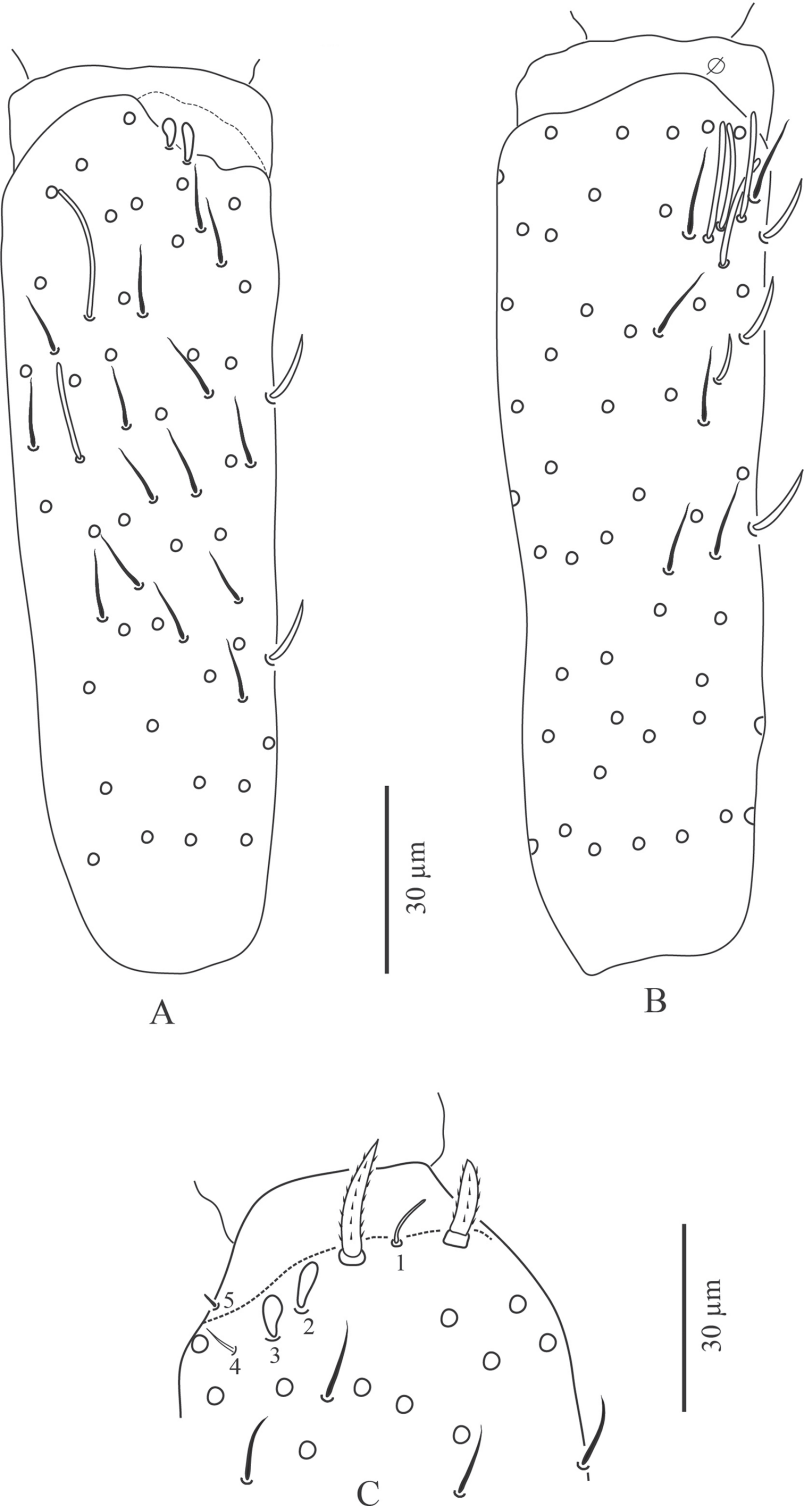
*Troglopedetes kae* sp. nov. continued **A** dorsal side of Ant. III, right side **B** ventral side of Ant. III, right side **C** distal organ of Ant. III, left side.

***Pseudopores*** (Figs 2A, 3B–D, 4B, 8A–D, 10B, 10D, E). Pseudopores present as round flat disks larger than mac sockets, on antennae, head and tergites. Dorsal pseudopore formula: 1–(2)/1, 1/1, 1, 1, 1+4 (Fig. 8A–D). On antenna, two psp detected ventro-distally on Ant. I, one ventro-distally on Ant. II and one ventro-distally on Ant. III (Figs 3B, D, 4B). On head, 1–(2) psp close to antennal basis (Fig. 2A). On legs, psp present externally on coxae (two for legs I and II and 2–(3) for leg III). On manubrium, two psp on the dorso-distal plaque (Fig. 10B, D, E), on dens (new location for *Troglopedetes*) two psp dorso-basally near the internal spine on each side (Fig. 10D, E).

***Mouthparts*.** Labral formula 4/5,5,4 (Fig. 2G); prelabral chaetae short, bent and ciliated, labral chaetae thinner, longer, smooth and acuminate, those of the distal row slightly shorter than those of the median row. Ventro-distal complex of labrum well differentiated, asymmetrical, with 2 distal combs (a larger one with 6–8 teeth on the left side, a smaller one with more than ten minute teeth on the right side) and an axial pair of sinuous tubules as in *Cyphoderopsis* (Jantarit et al. 2013) (Fig. 2F). Distal part of labrum not adorned with spines dorso-distally. Labial palp similar to that described by Fjellberg (1999) for *Troglopedetes* sp., with strong papillate chaetae. Indicative number of guards for each major papillate chaetae: A (0), B (5), C (0), D (4) and E (4); lateral process subcylindrical, reaching slightly above the apex of papilla E (Fig. 2C); five smooth proximal chaetae. Chaetae of labial basis as M1M2REL1l2, with M1, M2, E and L1 subequal and ciliated, R shorter than others and ciliated, l2 short, smooth and acuminate (Fig. 2I). Outer maxillary lobe with one papillate chaeta, one basal chaeta and two sublobal hairs, shorter than others (Fig. 2B). Maxillary head with a 3-toothed claw, several stout shortly ciliated lamellae not observed in detail and three thin elongate structures (two dorsally and one ventrally) (Fig. 2D, E). Mandible head strong, asymmetrical (left side with four teeth, right side with five teeth); molar plate with three strong pointed basal teeth, and other two or three inner distal teeth, identical in both mandibles (Fig. 2H).

***Ventral chaetotaxy of head*** (Fig. 2I). Head densely covered with oval scales (40–50 µm), postlabial chaetae along the linea ventralis as three mes anteriorly, one mac and an oblique line of five mes posteriorly on each side.

**Figure 5.**
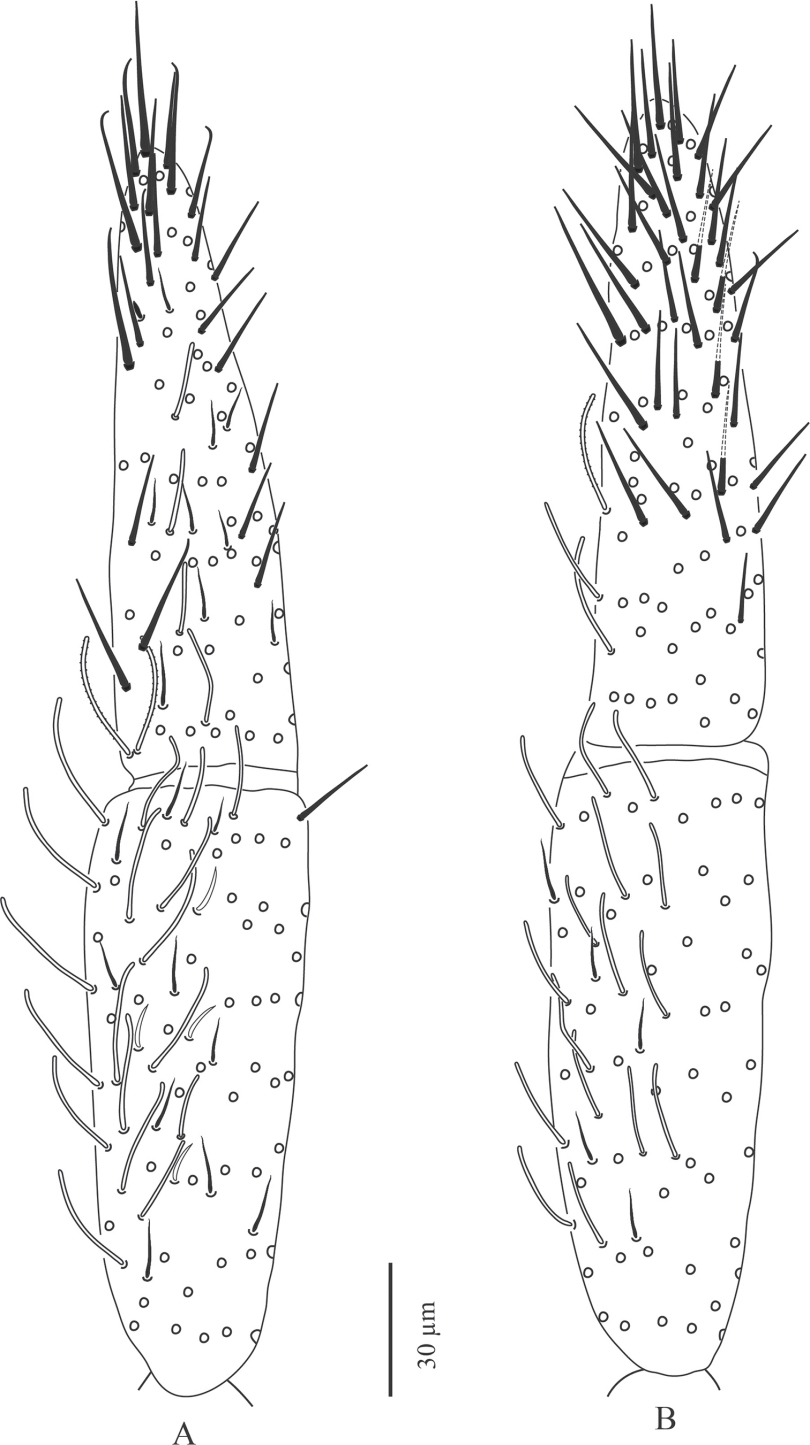
*Troglopedetes kae* sp. nov. continued **A** dorsal side of Ant. IV, right side **B** ventral side of Ant. IV, right side.

**Figure 6.**
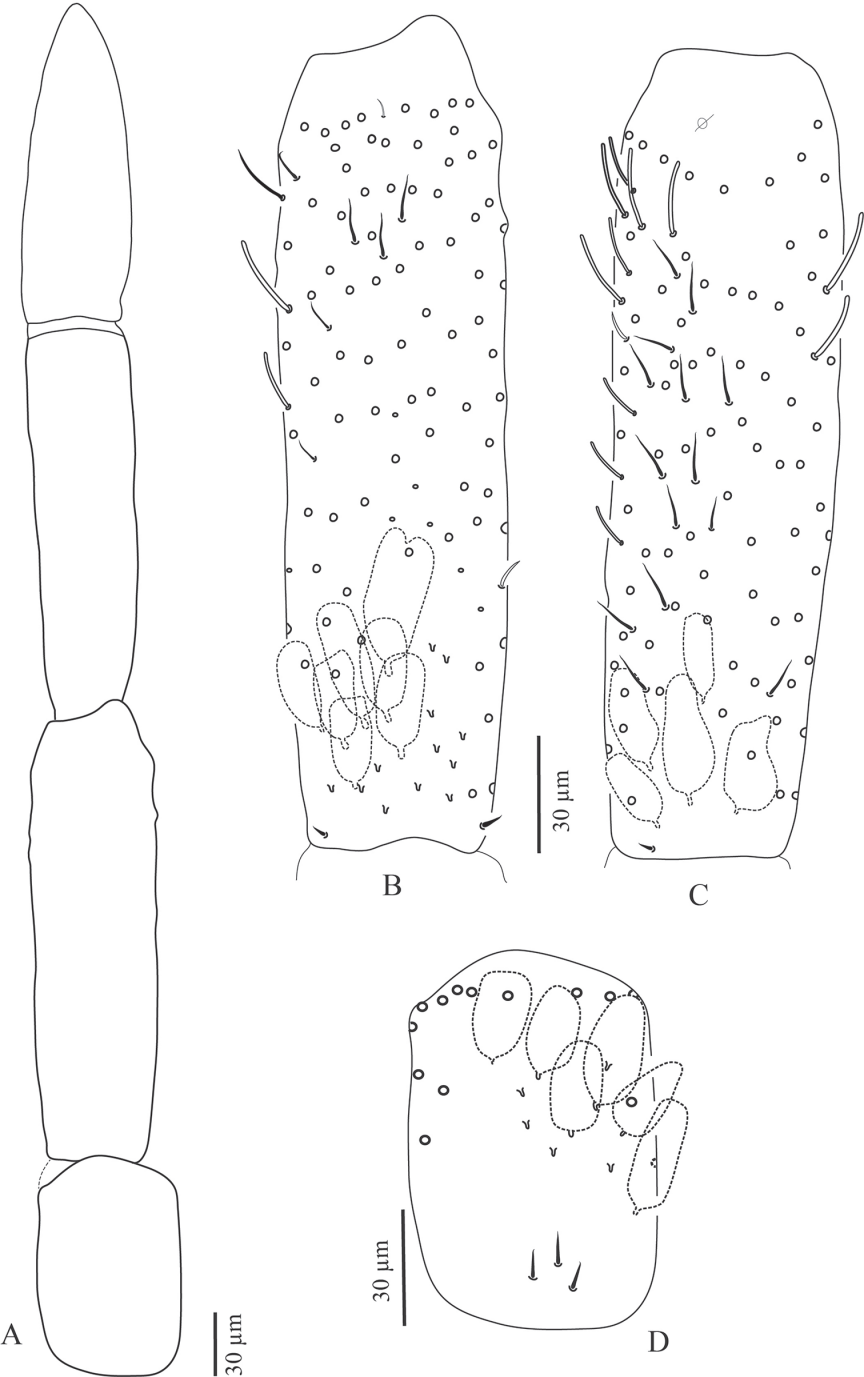
*Troglopedetes kae* sp. nov. continued **A** abnormal antenna with fusion of Ant. II and III, left side **B** dorsal side of fused Ant. II and III, left side **C** ventral side of fused Ant. II and III, left side **D** dorsal side of Ant. I, right side.

**Figure 7.**
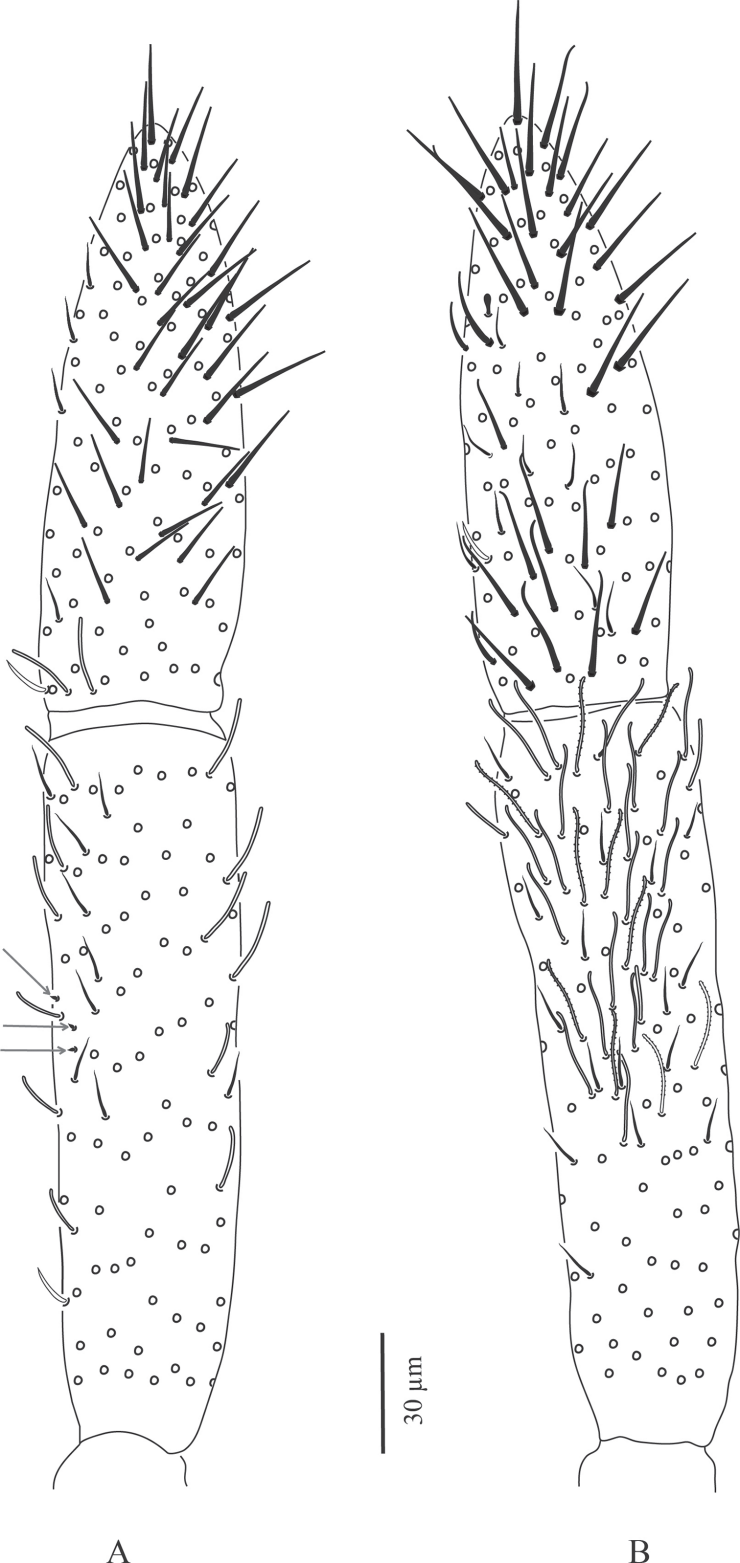
*Troglopedetes kae* sp. nov. continued **A** dorsal side of Ant. IV of a specimen with fused Ant. II and III, left side, arrows indicate new type of S-chaetae type 13 **B** ventral side of Ant. IV of a specimen with fused Ant. II and III, left side.

***Antennae*** (Figs 3–7). Antennae shorter than body, 2.2 × (n = 6) as long as cephalic diagonal. Ant. IV subdivided into two subequal segments, without apical bulb (Fig. 5). Lengths of antennal segments I-IV (IVa+IVb) as 1:1.9:1.3:2.5 (average, n = 6). Two other specimens with Ant. II and III fused (Figs 6, 7). Antennal chaetae (scales, five types of ordinary chaetae, 14 types of S-chaetae and subapical organite) described separately. Antennal scales oval, present dorsally only on Ant. I and II and ventrally on Ant. II, absent ventrally on Ant. I, and absent on Ant. III and IV (Figs 3A, C, D, 6B–D).

**Figure 8.**
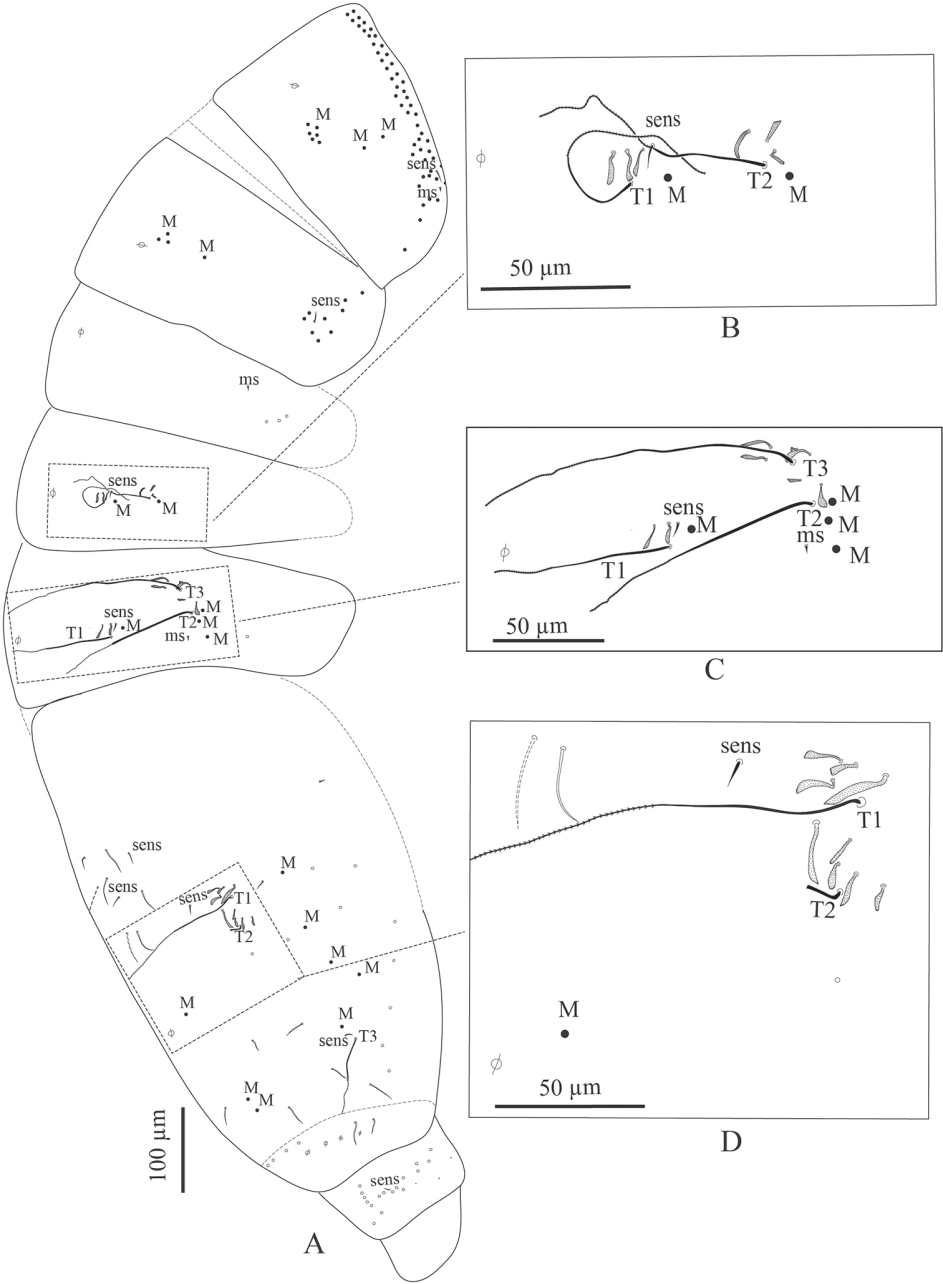
*Troglopedetes kae* sp. nov. continued **A** chaetotaxy of tergites **B** trichobothrial complexes of Abd. II **C** trichobothrial complexes of Abd. III **D** trichobothrial complex of Abd. IV.

***Body dorsal chaetotaxy*** (Figs 2A, 8A–D). Dorsal macrochaetae formula: 4,4/8,4/0,2,4,3 (Figs 2A, 8A). Trichobothrial pattern: 1/0, 0/0, 2, 3, 3 (Figs 2A, 8A). Trichobothrial complexes well developed with modified mes of various sizes (Fig. 8A–D) described below for each segment. The figured mes pattern is not complete.

Head with 12–13 peri-antennal mac in line on each side, with 4+4 central mac (chaetae A, B, E, F of Deharveng and Gers (1993), absence of the chaetae C, D and G, cephalic mes short, feebly serrated, equal, symmetrically arranged (not analysed). One lateral cephalic trichobothrium much shorter than closest mac on each side; suture zone not visible (Fig. 2A). Head dorsally densely covered with round to oval scales (20–35 µm). Body densely covered with oval scales (15–50 µm).

Th. II with a collar consisting of a few rows of mac along its anterior and antero-lateral margins, a compact group of six central mac on each side (“P3 complex” of Soto-Adames et al. (2014) and two antero-lateral mac; one antero-lateral ms; one antero-lateral sens; two or three short mic laterally, and a few others not counted centrally (Fig. 8A).

Th. III with four mac by side (a group of three central and one anterior to them), one sens at antero-lateral margins, and ca. eight or nine mac or long mes at lateral margins (Fig. 8A).

Abd. I without central mac, with one ms laterally on each side, and three mes laterally, mic not counted (Fig. 8A).

Abd. II with two tric on each side and six or seven modified mes around them (three or four around the internal tric and three near external tric), two mac (one near internal tric and one near external tric), one sens near internal tric (Fig. 8A, B), three mic (one close to internal tric and two close to external tric), others mes sockets internally visible, not counted.

Abd. III with three tric on each side (one internal, two external) and eight or nine modified mes around tric (two near internal tric, six or seven near the two external tric); four mac (one near internal tric and three near external tric); one sens anterior to internal tric and one ms posterior to the two external tric; several mes at lateral margins, not counted (Fig. 8A, C).

Abd. IV with three tric on each side (two antero-lateral, one postero-lateral) and ca. 7–9 modified mes around the two antero-lateral tric; postero-lateral tric without modified mes. Mac distributed as three central on each side (one antero-external to pseudopore, two anterior to posterior tergite margin), one near postero-lateral tric, and at least four external, mixed with many mes or smaller mac on lateral to posterior margins (not counted); probably three sens anteriorly; at least six S-like chaetae *sensu*Lukić et al. (2015) anteriorly, and several mes or S-like chaetae uniformly distributed (not counted); six serrated mes in line in the posterior row, four near axis and two along pseudopore line, from medium to short size (Fig. 8A, D).

Abd. V with only one sens detected on each side, and several ordinary chaetae from mes to mac, not counted (Fig. 8A). Abd. VI chaetotaxy not analysed.

**Figure 9.**
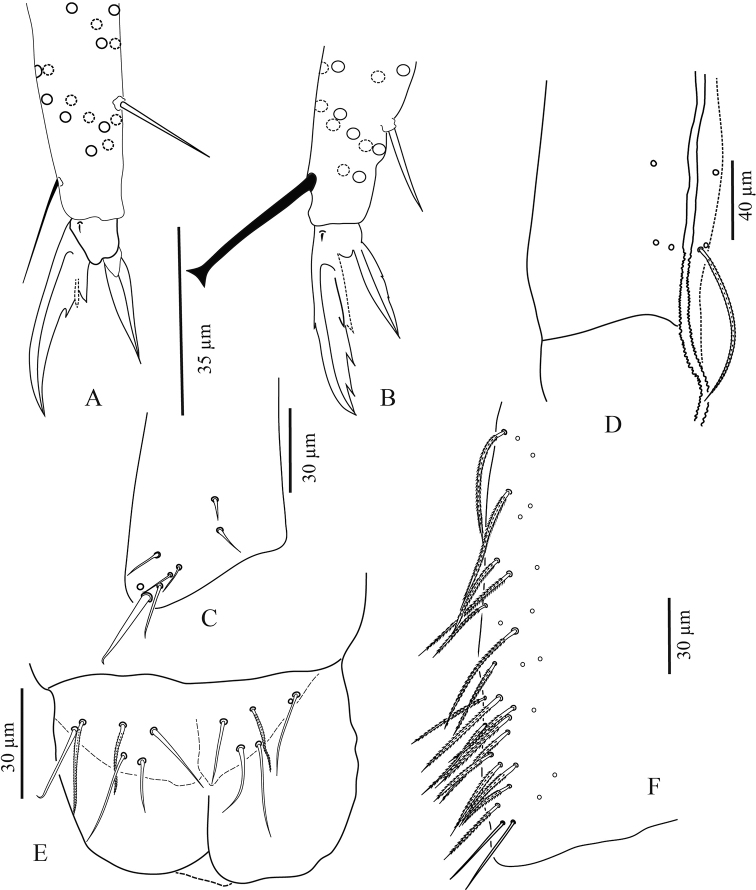
*Troglopedetes kae* sp. nov. continued **A** distal part of tibiotarsus III and claw complex with pointed tenent hair **B** distal part of tibiotarsus III and claw complex with clavate tenent hair **C** trochanteral organ **D** anterior side of ventral tube **E** lateral flap of ventral tube **F** posterior side of ventral tube.

***Legs*** (Fig. 9A–C). Legs long. Tita III as long as head diagonal, slightly longer than Tita I and II. Legs devoid of scales, mostly covered with ordinary ciliated chaetae of various length, from mes to mac. Trochanteral organ of leg III with eight smooth, straight, unequal, spiny chaetae (n = 1) (Fig. 9C). Tibiotarsus chaetotaxy mostly composed of strong ciliated-serrated mes, the basal ones longer and thicker (50–60 µm), slightly shorter distally (up to 15–20 µm). Distal row with 8–10 subequal ciliated mes and a dorso-apical tenent hair thin, smooth and acuminate on all tita; a ventro-distal strong smooth erected chaeta present on Tita III (Fig. 9A). Praetarsal mic minute (2.5–3 µm) (Fig. 9A). Unguis slender and relatively short (25–30 µm long, 6–7 µm wide at basis), 8–9 × shorter than tita, with one rather strong tooth at 30% of inner edge and a pair of inner basal teeth of unequal size; unguiculus pointed, narrow, lanceolate and elongate, ca. 0.6 × as long as claw, its external edge smooth (Fig. 9A).

***Ventral tube*** (Fig. 9D–F). Ventral tube ca. 4 × longer than wide, with 3+3 long serrated mac anteriorly (Fig. 9D) and six mes (two ciliated and four smooth) on each lateral flap (Fig. 9E); posteriorly with at least 31 long ciliated mes and two distal smooth mes (Fig. 9F).

**Figure 10.**
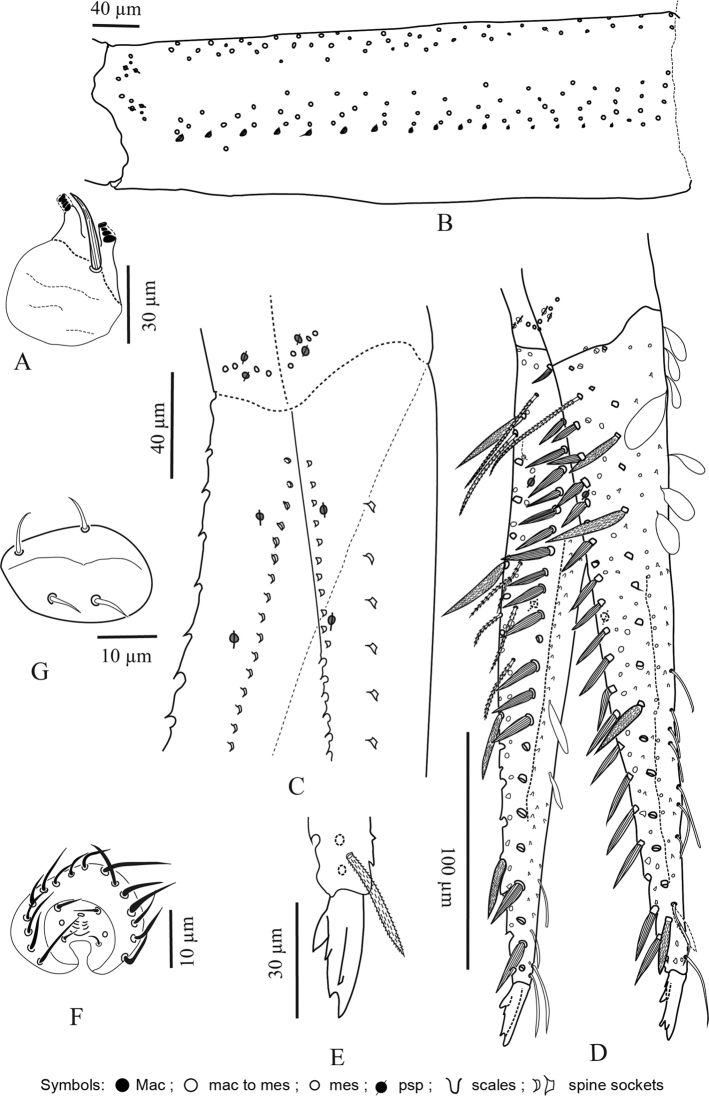
*Troglopedetes kae* sp. nov. continued **A** tenaculum **B** manubrium dorsal side **C** distal part of Manubrium and basal part of dens indicate the location of pseudopore **D** distal part of Manubrium and Mucrodens **E** mucro **F** male genital plate **G** female genital plate.

***Furcal complex*** (Fig. 10A–E). Tenaculum with four teeth on each ramus, of decreasing size from the basal to the distal one, on a prominent, irregular body, with a postero-basal strong, densely serrated, distally bent chaeta (Fig. 10A). Manubrium ca. 0.82 × (n = 8) shorter than mucrodens (mucro+dens). Manubrium dorsally with subequal ciliated mes (none smooth), irregularly arranged in three or four rows in two longitudinal stripes separated by a glabrous axial stripe, external row of chaetae distally with at least 7–11 long ciliated mes, dorso-distal plaque with 4+4 mes and 2+2 pseudopores (Fig. 10B). Ventrally, dense cover of round to oval (22–38 µm) and thin elongated scales (20–25 µm). Dens straight, elongate, hairy, slightly and progressively tapering, dorsally with two rows of spines, mixed with ciliated mes of various length, thickness and shape. Dorso-external row with 15–22 spines, dorso-internal row with 26–40 spines (asymmetries between dentes); external spines larger and less sclerotised than internal ones. Some short ciliated mes interspersed with spines in the external row; dorsally between the two rows of spines a mix of short and long ciliated mes, irregularly arranged in one row distally turning to three or four rows proximally; laterally, many short ciliated mes; dorso-distally, 3–(4) stronger ciliated mes; 2+2 psp on dorso-basally between the two rows of spine, sometimes inconspicuous (Fig. 10C, D). Dens ventrally entirely and densely scaled, the scales elongate (20–38 µm) (oval shape distally), arranged in short lines from 3–5 (distally) to 6–8 scales (proximally) (Fig. 10D). Mucro rather stout, short, 9.3–13.7 (average 12, n = 8) × shorter than dens (Fig. 10D, E), with five main teeth, the apical one blunt and strong, the subapical one acute and strong, a latero-distal one small and acute, and two dorso-basal, one minute and acute and one strong, acute and longer without toothlets basally (Fig. 10E).

***Genital plate*** (Fig. 10F, G). Male genital plate of the circinate type, with six genital mic and 16 circumgenital short, thin, smooth mes (Fig. 10F); female genital plate with 2+2 mic (Fig. 10G).

###### Ecology.

*Troglopedetes kae* sp. nov. is only known from a small chamber in the dark zone of a cave, accessible by a very low and narrow passage. Specimens were found as small populations in an oligotrophic habitat, i.e., on wall and ground with very humid and wet environment, without any trace of organic matter.

###### Etymology.

The species name is taken from the type locality (Tham Kae).

###### Remarks.

*Troglopedetes kae* sp. nov. has four medial head macrochaetae, three medial Abd. IV macrochaetae, one inner teeth of claw and mucro with five teeth. It is near *T. centralis* Deharveng & Gers, 1993 from a cave in Doi Chiang Dao, Chiang Mai province in the absence of eyes, chaetotaxy of labial basis and of outer maxillary lobe, dorsal macrochaetotaxy from head to Abd. IV, chaetotaxy of anterior side of ventral tube and claw morphology. However, *T. kae* sp. nov. differs from *T. centralis* by its smaller size (1.3–1.6 vs. 1.7–2.1 mm), body colour with spots of orange pigment (vs. white), longer antennae (0.5–0.6 × as long as body vs. 0.4), thinner claw with an internal tooth at 30 vs. 50–65% from the basis, chaetae on lateral flap of the ventral tube (6+6 vs. 7+7), higher ratio dens:mucro (9.3–13.7 vs. 8.8), and mucro with five teeth (vs. four).

In the same cave and same habitat, we found another morphotype with very different claw complex; thick and clavate tenent hair, two strong inner teeth on claw at 74% and 91% of inner edge, and external edge of unguiculus with two or three minute outer basal teeth, sometimes inconspicuous (Fig. 9B). However, material was not enough to describe it in detail.

The locality where *T. kae* sp. nov. was collected is located 400 km south of the Isthmus of Kra, i.e., more south than other described *Troglopedetes* from Thailand. The Isthmus of Kra is well-known to be a biogeographical transition zone between Indochinese and Sundaic fauna (Fig. 1). It was previously considered to be the southern distribution limit for the genus *Troglopedetes*, and the northern limit for the closely related genus *Cyphoderopsis* in Thailand (Deharveng and Gers 1993, Jantarit et al. 2013). This discovery provides evidence that the two genera may actually overlap in southern Thailand, and questions the role of the Isthmus of Kra as biogeographical barrier for cave fauna.

##### Troglopedetes meridionalis sp. nov.

Taxon classificationAnimaliaEntomobryomorphaParonellidae

04121417-9A49-5997-9648-44325892AF10

http://zoobank.org/9FA46FCC-4ED7-4DE0-9362-EC8425E63AAD

**Figure 11.**
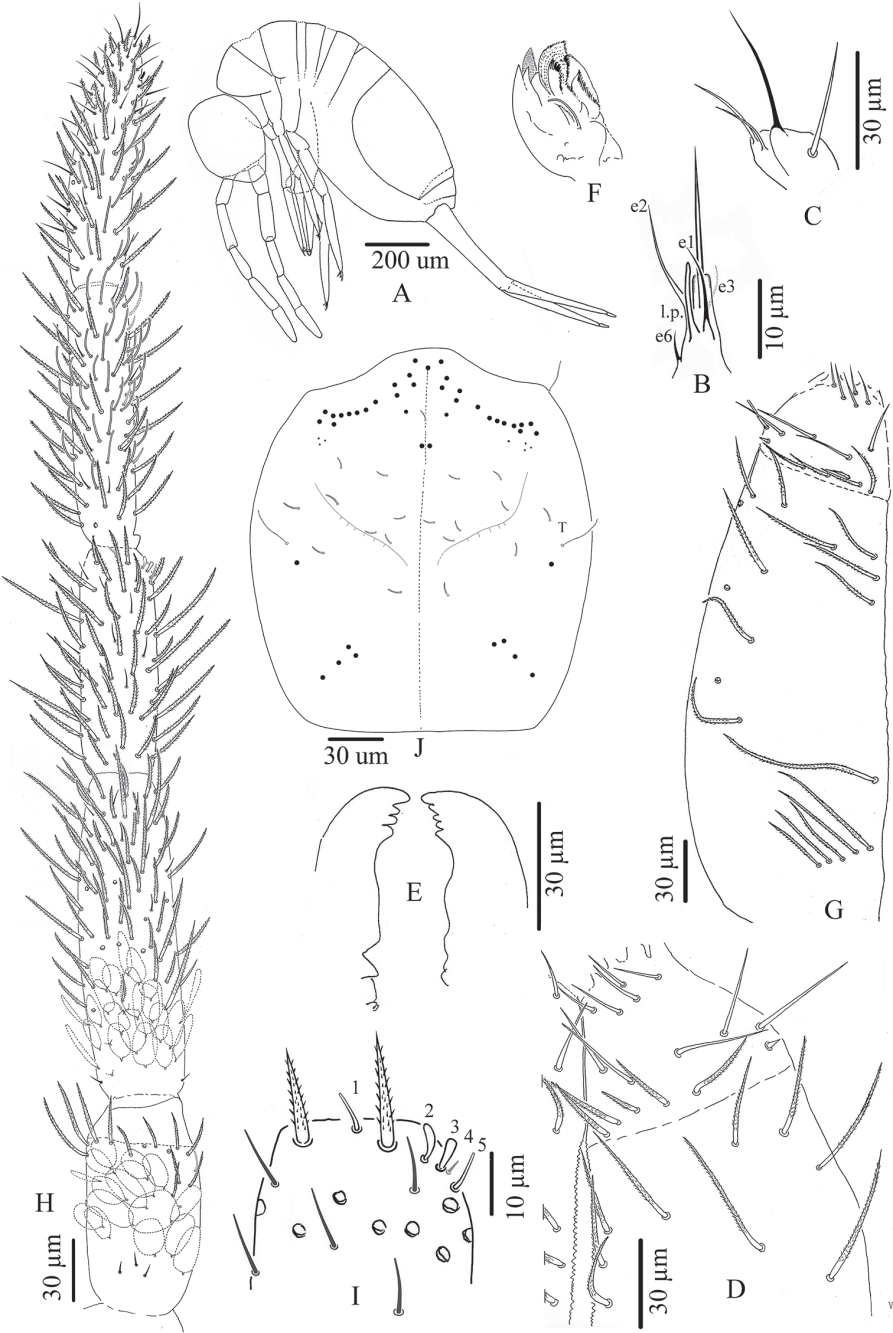
*Troglopedetes meridionalis* sp. nov. **A** habitus **B** labial palp of papillae E **C** outer maxillary lobe, right side **D** chaetotaxy of basal area of labium, right side **E** mandible **F** maxilla **G** ventral chaetotaxy of head, left side **H** antenna, right side **I** distal organ of Ant. III, right side **J** dorsal chaetotaxy of head.

**Figure 12.**
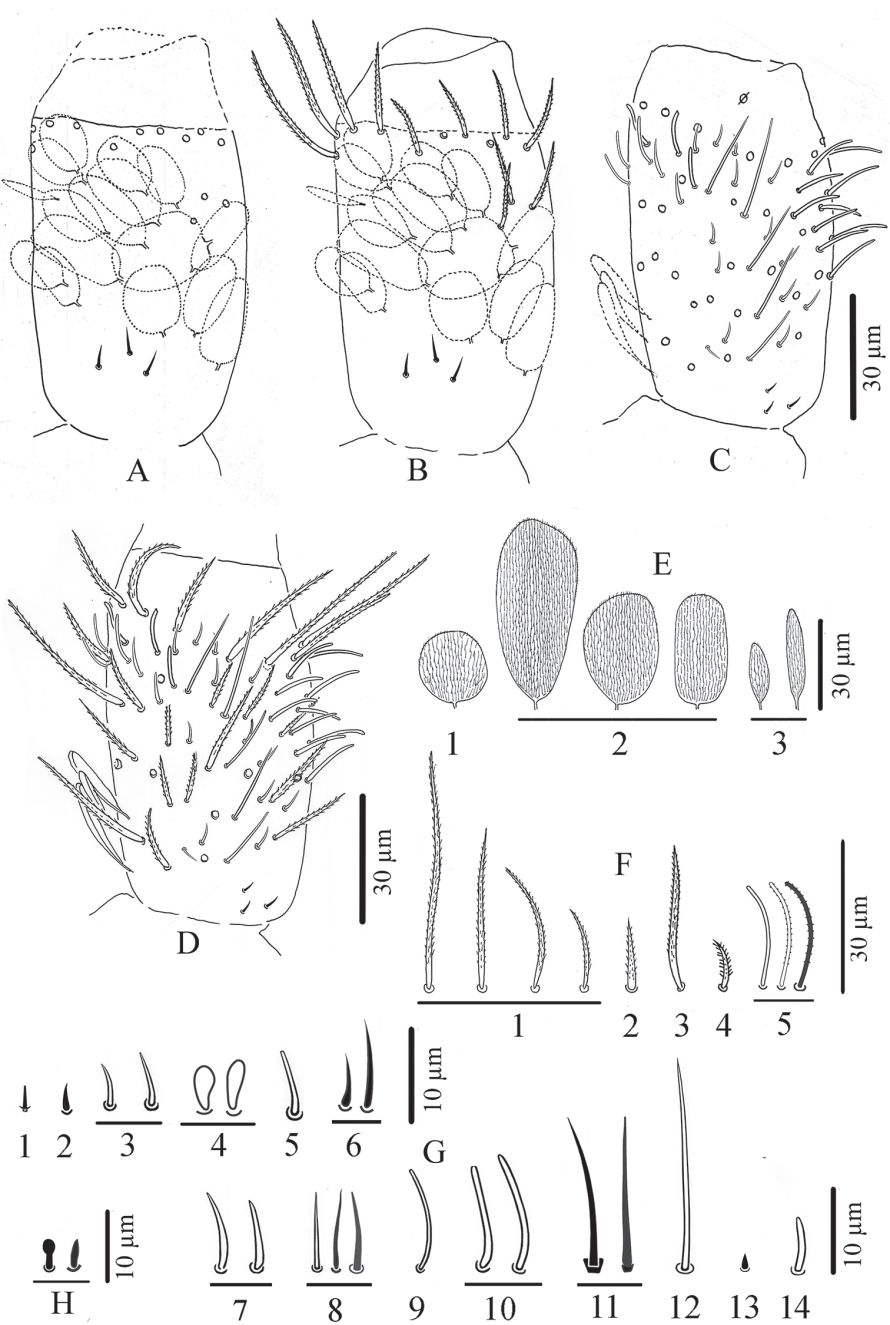
*Troglopedetes meridionalis* sp. nov. continued **A** dorsal side of Ant. I with scale and S-chaetae, right side **B** dorsal side of Ant. I with all chaetal types, right side **C** ventral side of Ant. I with scale and S-chaetae, right side **D** ventral side of Ant. I with all chaetal types, right side **E** type of scale **F** type of antennal ordinary chaetae **G** type of antennal S-chaetae **H** subapical organite of Ant. IV.

**Figure 13.**
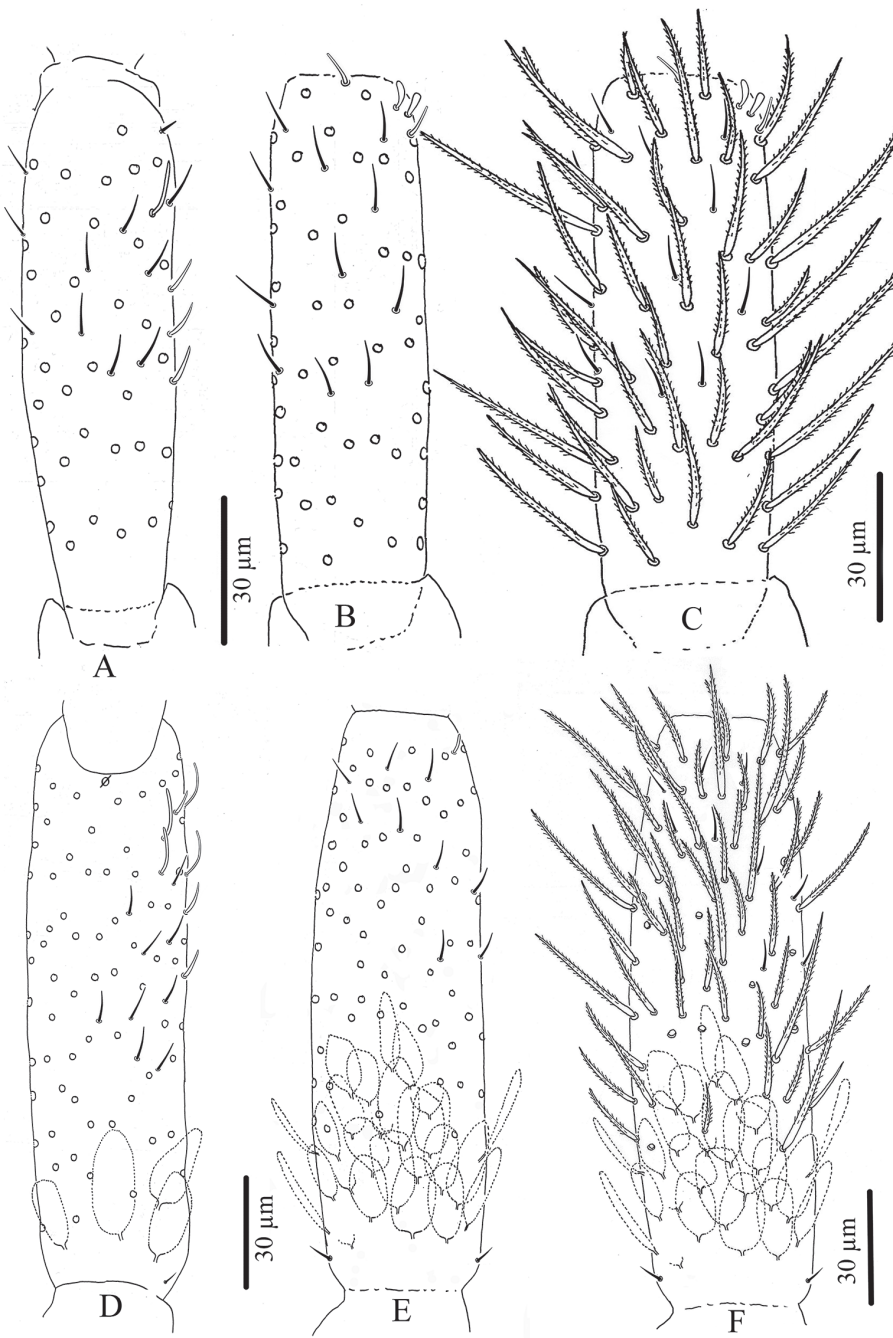
*Troglopedetes meridionalis* sp. nov. continued **A** ventral side of Ant. III with S-chaetae, right side **B** dorsal side of Ant. III with S-chaetae, right side **C** dorsal side of Ant. III with all chaetal types, right side **D** ventral side of Ant. II with scale and S-chaetae, right side **E** dorsal side of Ant. II with scale and S-chaetae, right side **F** dorsal side of Ant. II with all chaetal types, right side.

**Figure 14.**
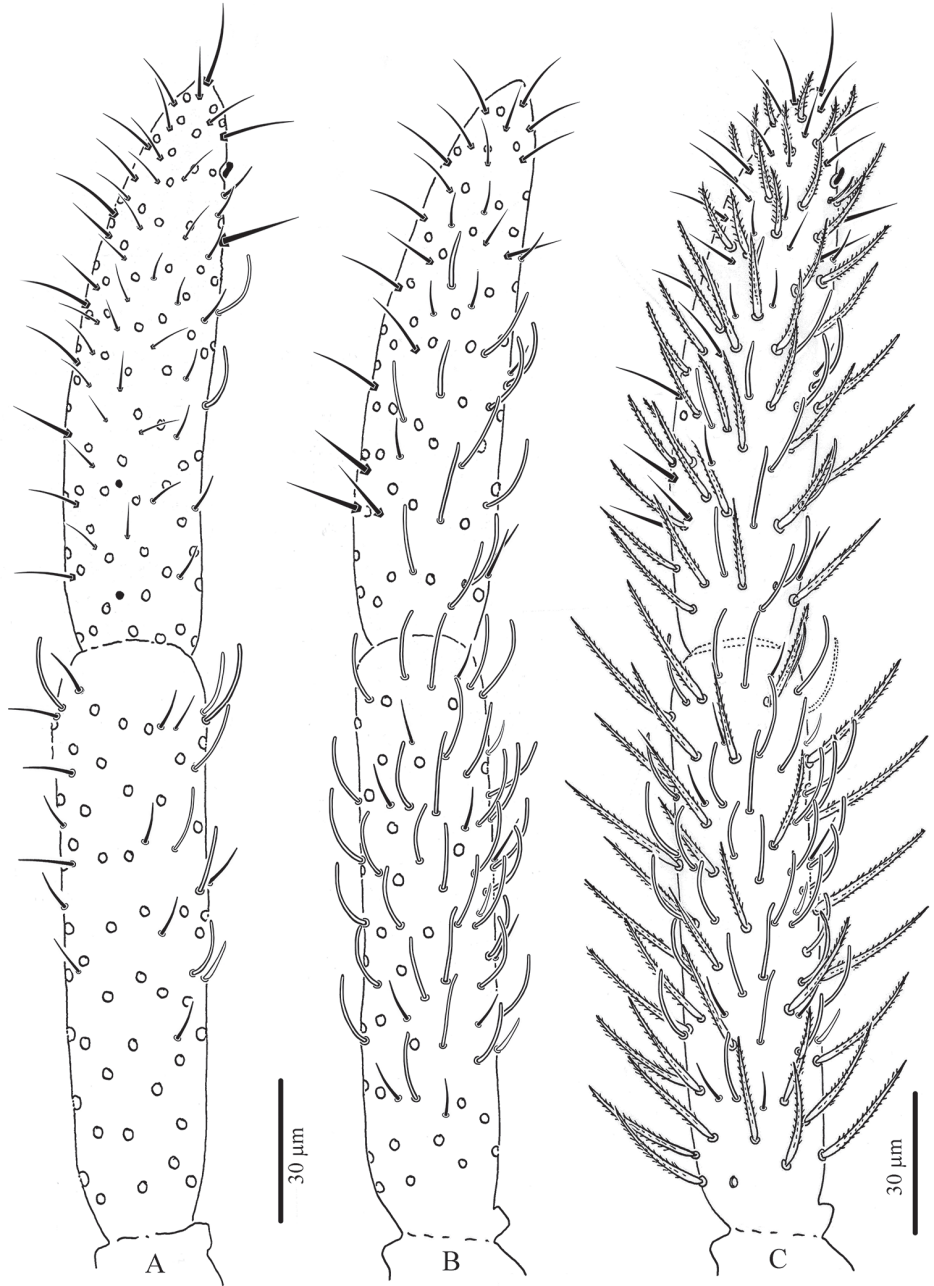
*Troglopedetes meridionalis* sp. nov. continued **A** ventral side of Ant. IV with S-chaetae, right side **B** dorsal side of Ant. IV with S-chaetae, right side **C** dorsal side of Ant. IV with all chaetal types, right side.

**Figure 15.**
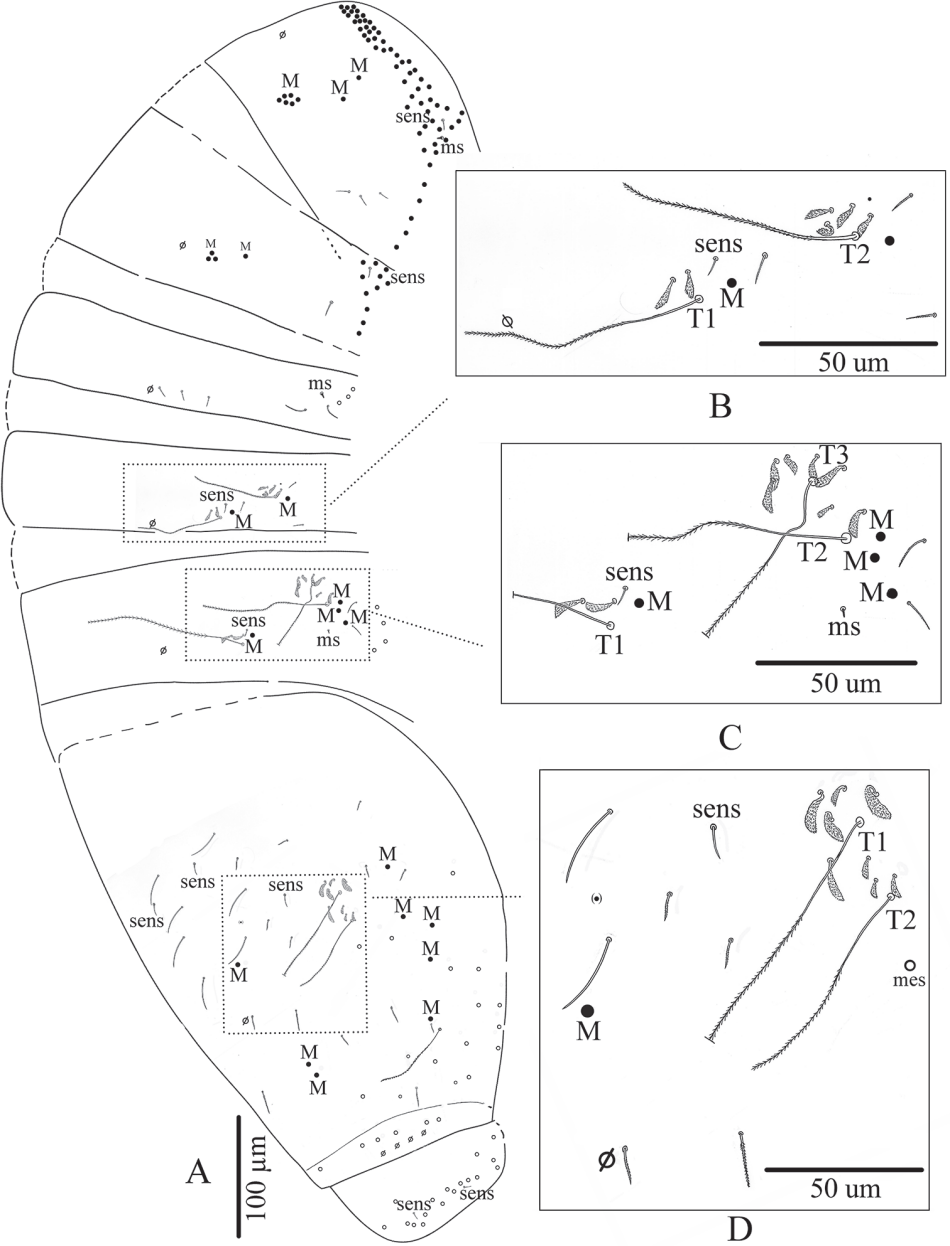
*Troglopedetes meridionalis* sp. nov. continued **A** chaetotaxy of tergites **B** trichobothrial complexes of Abd. II **C** trichobothrial complexes of Abd. III **D** trichobothrial complex of Abd. IV.

**Figure 16.**
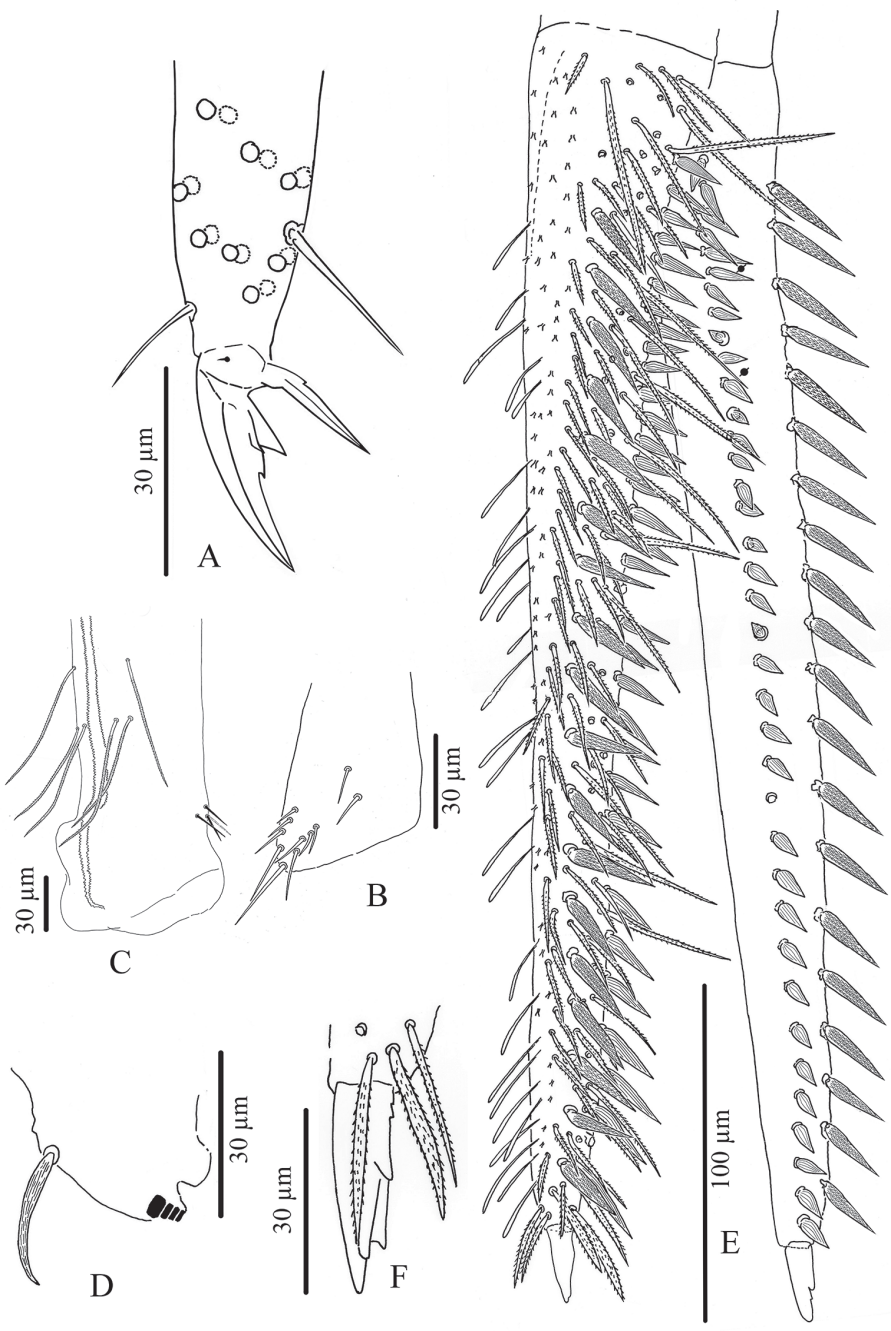
*Troglopedetes meridionalis* sp. nov. continued **A** distal part of tibiotarsus III and claw complex with pointed tenent hair **B** trochanteral organ **C** anterior side of ventral tube **D** tenaculum **E** mucrodens **F** enlargement of mucro and chaetae on tip of dens.

Figures 11 , 12 , 13 , 14 , 15 , 16

###### Type material.

Holotype male and four paratypes (one female, three subadults) on slides. Thailand: Chumphon Province: Lang Suan district, Tham Don Non (Tapan), 9°54'14"N, 99°02'41"E, ca 60 m a.s.l., 25 Jul. 2015, S. Jantarit leg., dark zone of cave, by aspirator (sample # THA_SJ_CPN01). Holotype and two paratypes deposited in NHM-PSU. Two paratypes deposited in MNHN. Measurements of holotype in Table 2.

**Table 2.** *Troglopedetes meridionalis* sp. nov., measurements in µm (from holotype).

Head		Body		Appendages	
Ant. I	78	Th. II	137	Man	345
Ant. II	150	Th. III	114	Dens	340
Ant. III	125	Abd. I	62	Mucro	30
Ant. IVa	125	Abd. II	76	Furca	715
Ant. IVb	125	Abd. III	110	Claw I	27
Ant.	603	Abd. IV	400	Claw II	27
Head	300	Abd. V	62	Claw III	27
		Abd. VI	40		
		Body	1,301		

###### Description.

***Habitus*.** Slightly troglomorphic, slender, with elongate legs, furca and antennae (Figs 1, 11A). Body length 1.3–1.5 mm. Fourth abdominal segment 3 × as long as the third one along dorsal axis. Furca well developed, ca. 2 × shorter than body length. Body colour white with spots of orange pigment. Eyes absent, no ocular patch.

***Chaetal types*.** Four types of chaetae on somites, appendages (except antennae) and mouthparts: scales, present on antennae I and II, head, body and furca, absent on legs and ventral tube; ordinary chaetae on all body parts; S-chaetae and trichobothria on tergites; hairs devoid of sockets on outer maxillary lobe. Chaetal types on antennae are much more diverse and described separately further.

***Pseudopores*** (Figs 12C, 13D, 15A, B, D). Pseudopores present as round flat disks larger than mac sockets, on antennae, head and tergites. Dorsal pseudopore formula: 1–(2)/1, 1/1, 1, 1, 1+4 (Figs 11J, 15A). On antenna, 1–(2) psp detected ventro-distally on Ant. I., one ventro-distally on Ant. II and one ventro-distally on Ant. III, sometimes not clearly seen (Figs 12C, 13D). On head, 1–(2) psp close to antennal basis. On legs, psp present externally on coxae (two for legs I and II and 2–(3) for leg III). On manubrium, two psp on each dorso-distal plaque, on dens (new location for *Troglopedetes*) two psp dorso-basally near the internal spine on each side (Fig. 16E).

***Mouthparts*.** Labral formula 4/5,5,4; prelabral chaetae short, bent and ciliated, labral chaetae thinner, smooth and acuminate, those of the distal row slightly shorter than those of the median row. Ventro-distal complex of labrum well differentiated, asymmetrical, with two distal combs (a larger one with 6–8 teeth on the left side, a smaller one with more than ten minute teeth on the right side) and an axial pair of sinuous tubules. Distal part of labrum not adorned with spines dorso-distally. Labial palp similar to that of *T. kae* sp. nov. (Fig. 11B). Chaetae of labial basis as M1M2REL1l2. Chaetae M1, M2, E and L1 subequal and ciliated, R shorter than others and ciliated, l2 short, smooth and acuminate (Fig. 11D, G). Outer maxillary lobe with one papillate chaeta, one basal chaeta and two sublobal hairs, shorter than others (Fig. 11C). Maxillary head with a 3-toothed claw, several stout shortly ciliated lamellae not detailed here; special structures present on the maxilla head, i.e., a thin elongate structure, arising from the basis of the maxilla head and reaching claw basis; close to it a spiny structure and a thin structure in an opposite side (Fig. 11F). Mandible heads strong, asymmetrical (left side with four teeth, right side with five teeth); molar plate with three strong pointed basal teeth, and other two or three inner distal teeth, identical in both mandibles (Fig. 11E).

***Ventral chaetotaxy of head*** (Fig. 11D, G). Head densely covered with oval scales (40–50 µm), postlabial chaetae along the linea ventralis as three mes anteriorly, one mac and one oblique line of four or five mes posteriorly on each side.

***Antennae*** (Figs 11H–I, 12A–D, 13A–F, 14A–C). Antennae shorter than body, ca. 2 × (n = 3) as long as cephalic diagonal. Ant. IV subdivided into two subequal segments, without apical bulb. Lengths of antennal segments I to (IVa+IVb) as 1:1.8:1.5:2.9 (average, n = 3). Antennal chaetae (scales, five types of ordinary chaetae, 13 types of S-chaetae and subapical organite) described separately. Antennal scales oval, present dorsally only on Ant. I and II and ventrally on Ant. II, absent ventrally on Ant. I, and absent on Ant. III and IV (Figs 11H, 12A–D, 13D–F).

***Dorsal chaetotaxy*** (Figs 11J, 15A, B, D). Dorsal macrochaetae formula: 1,4/8,4/0,2,4,3 (Figs 11J, 15A). Trichobothrial pattern: 1/0, 0/0, 2, 3, 3 (Figs 11J, 15A, B, D). Trichobothrial complexes well developed with modified mes of various sizes (Fig. 15A–D) as described below for each segment. The mes pattern is not complete. Head with 12 or 13 peri-antennal mac in line on each side; with 1+1 central mac (chaetae A of Deharveng and Gers 1993), chaetae B-F absent, at least 9+9 cephalic mes short, feebly serrated, equal in size, symmetrically arranged (Fig. 11J). One lateral cephalic trichobothria much shorter than closest mac on each side; suture zone visible, without associated mac (Fig. 11J). Head dorsally densely covered with round to oval scales (20–35 µm). Body densely covered with oval scales (15–50 µm).

Th. II collar consisting of a few rows of mac along its anterior and antero-lateral margins, a compact group of six central mac on each side (“P3 complex”) and two antero-lateral mac; one antero-lateral ms; one antero-lateral sens; two or three short mic laterally, and a few others not counted centrally (Fig. 15A).

Th. III with four mac on each side (a group of three central and one anterior to them); one sens at antero-lateral margins; one mic laterally; and ca. 11+11 mac or long mes at lateral margins (Fig. 15A).

Abd. I without central mac, with one ms laterally on each side; two or three mic arranged in line externally to pseudopore and two larger lateral mic; three mes laterally (Fig. 15A).

Abd. II with two tric on each side and six or seven modified mes around them (two around the internal tric and four or five near external tric), two mac (one near internal tric and one near external tric), one sens near internal tric; three mic (one close to internal tric and two close to external tric), others mes sockets internally visible, not counted (Fig. 15A, B).

Abd. III with three tric on each side (one internal, two external) and nine or ten modified mes around tric (two near internal tric and seven or eight near the two external tric); four mac (one near internal tric and three near external tric); one sens anterior to internal tric and one ms posterior to the two external tric; two mic/mes external to the external mac; several mes at lateral margins, not counted (Fig. 15A, C).

Abd. IV with three tric on each side (two antero-lateral, one postero-lateral); and ca. 6–9 modified mes around the two antero-lateral tric; postero-lateral tric without modified mes. Mac distributed as three central on each side (one antero-external to pseudopore, two anterior to posterior tergite margin), one near postero-lateral tric, and four or five external, mixed with many mes or smaller mac on lateral to posterior margins (not counted); probably three sens anteriorly; at least seven S-like chaetae *sensu*Lukić et al. (2015), and several mes or S-like chaetae uniformly distributed (not counted); six serrated mes in line in the posterior row (one near axis and five along pseudopore line, from medium to short) (Fig. 15A, D).

Abd. V with only two sens detected on each side, and several ordinary chaetae from mes to mac in size (Fig. 15A). Abd. VI chaetotaxy not analysed.

***Legs*** (Fig. 16A, B). Legs long; Tita III as long as head diagonal, slightly longer than Tita I and II. Legs devoid of scales, mostly covered with ordinary ciliated chaetae of various length, from mes to mac. Trochanteral organ of leg III with 11–13 smooth, straight, unequal spiny chaetae (Fig. 16B). Tibiotarsus chaetotaxy mostly composed of strong ciliated-serrated mes, basal ones longer and thicker (60 µm), the distal ones slightly shorter (up to 15–20 µm). Distal row with ten subequal ciliated mes and a dorso-apical tenent hair thin, smooth and acuminate on all tita; a ventro-distal strong smooth erected chaeta present on Tita III (Fig. 16A). Praetarsal mic minute (2.5–3 µm, Fig. 16A). Unguis slender and relatively short (27–30 µm long, 6–7 µm wide at basis), 8–9 × shorter than tita; with one rather strong tooth at 50–55% of inner edge and a pair of inner basal teeth of unequal size (Fig. 16A). Unguiculus acuminate, 0.6 × as long as claw, its external edge with a minute outer basal tooth, sometimes inconspicuous (Fig. 16A).

***Ventral tube*** (Fig. 16C). Ventral tube 4 × longer than wide; with 3+3 long serrated mac anteriorly and six mes (two ciliated and four smooth) on each lateral flap (Fig. 16C); posteriorly with many long ciliated mes, not suitable for observation in available specimens.

***Furcal complex*** (Fig. 16D–F). Tenaculum with four large teeth on each ramus, of decreasing size from the basal to the distal one, on a prominent, irregular body, with a postero-basal strong, densely serrated, distally bent chaeta (Fig. 16D). Manubrium slightly shorter than mucrodens (ca. 0.95 ×). Manubrium dorsally with subequal ciliated mes (none smooth) irregularly arranged in 3–4 rows in two longitudinal stripes separated by a glabrous axial stripe, external row of chaetae distally with at least 5–7 long ciliated mes, dorso-distal plaque with 4+4 mes and 2+2 pseudopores. Ventrally, dense cover of round to oval (20–38 µm) and thin elongated scales (20–25 µm).

Dens straight, elongate, hairy, slightly and progressively tapering, dorsally with two rows of spines, mixed with ciliated mes of various length, thickness and shape. Dorso-external row with 17–20 subequal spines, dorso-internal row with 30–37 subequal spines (asymmetries between dentes); external spines larger and less sclerotised than internal ones. Short ciliated mes interspersed with spines in the external row; dorsally between the two rows of spines a mix of short and long ciliated mes, irregularly arranged in one row distally turning to 3–4 rows proximally; laterally, many short ciliated mes; dorso-distally, 3–(4) stronger ciliated mes; 2+2 psp on dorso-basally between the two rows of spine, sometimes inconspicuous. Dens ventrally entirely and densely scaled, scales elongate (20–38 µm), arranged in short lines from 3–5 (distally) to 6–8 scales (proximally) (Fig. 16E).

Mucro rather stout, short, 12–15 × shorter than dens (Fig. 16E, F), with four main teeth, the apical one blunt and strong, the two subapical ones smaller, the dorso-basal one slightly longer with one or two toothlets basally (Fig. 16F).

***Genital plate*.** Genital plate not seen.

###### Ecology.

*Troglopedetes meridionalis* sp. nov. was found in small populations in the dark zone of a cave, foraging on a small patch of old and humid bat guano.

###### Etymology.

The name of the species, *meridionalis*, means southern in Latin, referring to the location of the species in the southern part of peninsular Thailand.

###### Remarks.

*Troglopedetes meridionalis* sp. nov. is the only species of the genus with one medial head macrochaeta. It is similar to *T. convergens* Deharveng & Gers, 1993 from a cave of Ratchaburi province in the absence of eyes, two rows of dental spines, labial basis chaetotaxy, similar dorsal chaetotaxy from head to Abd. IV, anterior ventral tube chaetotaxy, and claw morphology. It can be distinguished by the combination of characters listed in Tables 3A and 3B, in particular the absence of the chaetae “E” on head, larger size (1.3–1.5 vs. 0.95–1.3 mm), antennae relatively shorter (0.35–0.4 vs. 0.45–0.5 × as long as body), tenent hair acuminate vs. usually clavate, each lateral flap of ventral tube with six chaetae (vs. seven), dental spines of the internal row more numerous (30–37 vs. 19–23) and higher ratio of dens:mucro (12–15 vs. 9).

The pair of mac immediately ahead A and that ahead the uneven anterior mac on head are not figured in Deharveng and Gers (1993), as they were not considered as mac, but long mes. Their sockets in the new species are smaller than those of mac, but clearly marked.

**Table 3A.** Summary of main general and head characters of Thai *Troglopedetes* (N = northern Thailand, W = western Thailand, S = southern Thailand).

Species/Characters	Length (mm)	Eyes	Colour	Ant.: Body	Labial basis	Sublobal hairs	Central mac on head	Mac on head	Locality (province)
*T. calvus*	0.90–1.37	0	white	0.6	M1M2REL1l2	2	0	–	Kanchanaburi (W)
*T. centralis*	1.7–2.1	0	white	0.4	M1M2REL1l2	2	4+4	A, B, E, F	Chiang Mai (N)
*T. convergens*	0.95–1.3	0	white	0.45–0.5	M1M2REL1l2	2	2+2	A, E	Ratchaburi (W)
*T. dispersus*	1.3–1.4	0	white with red pigment	0.6	M1M2Re(E)L1l2	2	3+3	A, C, E	Kanchanaburi (W)
*T. fredstonei*	1.4–2.1	0	white with red pigment	0.5	M1M2REL1l2	2	5+5	A, B, C, E, F	Chiang Mai (N)
*T. kae* sp. nov.	1.3–1.6	0	white with orange pigment	0.46	M1M2REL1l2	2	4+4	A, B, E, F	Satun (S)
*T. leclerci*	0.7–1.0	3+3	white	0.44	M1M2REL1l2	2	7+7	A, B, C, D, E, F, G	Chiang Mai (N)
*T. longicornis*	1.8–2.2	0	white	0.8	M1M2ReL1l2	2	5–6+5–6	A, B, C, D, E, (F)	Mae Hong Son (N)
*T. maffrei*	1.3–1.75	0	white	0.4	M1M2ReL1l2	1	7+7	A, B, C, D, E, F, G	Mae Hong Son (N)
*T. maungonensis*	1.1–1.2	0	white	0.5	M1M2REL1l2	1	7+7	A, B, C, D, E, F, G	Chiang Mai (N)
*T. meridionalis* sp. nov.	1.3–1.5	0	white with orange pigment	0.46	M1M2REL1l2	2	1+1	A	Chumphon (S)
*T. microps*	1.5–2.0	1–2+1–2	white	0.6	M1M2REL1l2	1	4–5+4–5	A, B, C, E, (F)	Chiang Mai (N)
*T. multispinosus*	1.8–2.2	0	white, red eye patch	0.9	M1M2ReL1l2	2	3+3	A, B, E	Chiang Rai (N)
*T. paucisetosus*	0.85–1.06	0	white with some red pigment	0.6	M1M2REL1l2	2	1–2+1–2	A, (E)	Prachuap Khiri Khan (W)

**Table 3B.** Summary of main characters of body and appendages of Thai *Troglopedetes* (N = northern Thailand, W = western Thailand, S = southern Thailand, ? = no information).

Species/ Characters	Central Mac on Th. II	Central Mac on Abd. IV	Claw (inner teeth)	Position of inner teeth	Tenent hair	Lateral flaps of VT	Internal row of spine	Dens: mucro	Locality (province)
*T. calvus*	8+8	3+3	2	65%, 85%	capitate	7+7	14–32	8.5	Kanchanaburi (W)
*T. centralis*	8+8	3+3	1	50–65%	pointed	7+7	37–42	8.8	Chiang Mai (N)
*T. convergens*	8+8	3+3	1	55%	capitate	7+7	19–23	9	Ratchaburi (W)
*T. dispersus*	8+8	3+3	1(2)	50%–(80%)	pointed or capitate	7+7	25–29	8.5	Kanchanaburi (W)
*T. fredstonei*	8+8	3+3	1	58–60%	pointed or capitate	7+7	?	15	Chiang Mai (N)
*T. kae* sp. nov.	8+8	3+3	1	30–35%	pointed	6+6	26–33	9.3–13.7	Satun (S)
*T. leclerci*	8+8	2+2	1	50%	pointed or feebly capitate	6+6	18	5–6	Chiang Mai (N)
*T. longicornis*	8+8	3+3	1	30–45%	pointed	7+7	29–36	13.1	Mae Hong Son (N)
*T. maffrei*	9+9	3+3	2	65, 75–85%	pointed or capitate	6+6	21–28	9.4	Mae Hong Son (N)
*T. maungonensis*	9+9	3+3	2	65, 85%	capitate	?	27–30	9.7	Chiang Mai (N)
*T. meridionalis* sp. nov.	8+8	3+3	1	50–55%	pointed	6+6	30–37	12–15	Chumphon (S)
*T. microps*	8+8	3+3	1	65%	capitate	7+7	36–42	13.6	Chiang Mai (N)
*T. multispinosus*	8+8	3+3	1	35%	pointed	7+7	34–41	12.7	Chiang Rai (N)
*T. paucisetosus*	8+8	2+2	2	65, 80%	capitate	7+7	18–24	9.6	Prachuap Khiri Khan (W)

### Diversity of antennal chaetae in *T. kae* sp. nov. and *T. meridionalis* sp. nov.

The work presented below is an attempt to build a comprehensive list of phanere types found on the antennae of an Entomobryoidea, to describe them morphologically, and to explore their distribution on antennal surface. Antennae bears many useful taxonomic and phylogenetic characters in Poduromorpha and has been the object of many studies (Deharveng 1981). In Entomobryoidea, antennal chaetotaxy is much more complex and variable, and has never been thoroughly explored for this reason. Only a few characters easy to observe are routinely used in taxonomy. Three recent papers, however (Lukić et al. 2015, 2018; Jantarit and Sangsiri 2020) provide detailed information on the chaetal investiture of *Verhoeffiella* and *Alloscopus* species (family Entomobryidae). Here, we carried out a detailed analysis of antennal chaetae of the adult of the two new *Troglopedetes* species described above, one female (normal antennae, Figs 3–5) and one female (fused antennae, Figs 6, 7) for *T. kae* sp. nov. and a male of *T. meridionalis* sp. nov. (Figs 11H, 12A–D, 13A–F, 14A–C).

#### Types of phaneres

There are five main types of phaneres (*sensu*Deharveng 1983) in *Troglopedetes*, like in all scaled Entomobryoidea: scales, ordinary chaetae (including spines), trichobothria, S-chaetae and subapical organite of Ant. IV. All, except trichobothria, are present on the antennae. We list phanere types that we recognise within each of these broad categories, mentioning putative homologies from the literature, and we summarise their arrangement pattern on each antennal segment.

#### Scales

Three types of scales were recognised in Thai *Troglopedetes*, but only one type was found on antennae of *T. kae* sp. nov. All scales are roundish, oval, variable in size on different organs, devoid of ridges but adorned by a dense and homogeneous cover of minute spicules (Fig. 12E).

Type 1 – round shape (20–30 µm long) on body and furca (Fig. 12E1).

Type 2 – oval shape (15–50 µm × 7–30 µm) on antennae, body and furca (Fig. 12E2).

Type 3 – thin, elongated (20–40 µm × 5–8 µm) on ventral side of dens (Fig. 12E3).

Antennal scales are of type 2 (25–35 µm long), oval, present dorsally only on Ant. I and II and ventrally on Ant. II, absent ventrally on Ant. I, and absent on Ant. III and IV (Figs 3A, C, D, 6B–D, 11H, 12A–D, 13D–F).

#### Ordinary chaetae

Ordinary chaetae are the most numerous chaetae on the body and appendages of *Troglopedetes*. They are well-diversified on antennae (Fig. 12F), where five types have been recognised. In *Verhoeffiella* and *Alloscopus*, Lukić et al. (2015) and Jantarit and Sangsiri (2020) recognised three types of ordinary chaetae on the antennae.

- Normal mes: serrated or ciliated (10–70 µm), of various thickness, shape and size, present on all antennal segments (Fig. 12F1).
- Short tapering mes: thicker than normal mes, straight or weakly bent, ciliated (15–18 µm), present dorso-distally on Ant. III (Figs 4C, 11I, 12F2).
- Long thick mes: thicker than normal mes, long and rather broadly ciliated (30–35 µm), present dorso-proximally on Ant. I (Figs 11H, 12B, 12F3).
- Short thick mes: thicker than normal mes, not clearly tapering, bent, ciliated (≈ 10 µm), limited to the tip of Ant. IV (Figs 11H, 14C, 12F4).
- Long, subcylindrical, hyaline, weakly bent and finely serrated mes (> 15 µm) (sometimes looking dark) mostly present on both dorsal and ventral sides of Ant. IV (Figs 5A, B, 7A, B, 12F5, 14A–C), that may appear smooth and qualified of S-chaetae under light microscope.

#### S-chaetae

S-chaetae (*sensu*Deharveng 1983) are present on all antennal segments, with a variety of thicknesses, shapes, and sizes (from mic to mes, 2–30 µm). The antennae of our species were carefully examined under light microscope. As a result, 14 types of S-chaetae were recognised in *T. kae* sp. nov. and 13 types in *T. meridionalis* sp. nov. based on morphology: thickness, length, bending (straight vs. bent), orientation (erected vs. oblique to the integument), shape (cylindrical vs. tapering vs. foliaceous), tip morphology (pointed vs. blunt), and opacity (hyaline vs. dark) (Fig. 12G). When possible, they were homologised with those of *Verhoeffiella* (Lukić et al. 2015) and *Alloscopus* (Jantarit & Sangsiri, 2020) as described below.

Type 1–minute mic, thin, pointed and dark (3–4 µm) (Fig. 12G1); corresponding to antennal S-chaetae type j *sensu*Lukić et al. (2015) and Jantarit and Sangsiri (2020).

Type 2–short mic, thin, usually bent and dark mic (5–6 µm) (Fig. 12G2).

Type 3–short mic, thin, rather curved apically and hyaline (5–6 µm) (Fig. 12G3).

Type 4–short, hyaline and swollen mic (foliaceous sens) (5–6 µm) (Fig. 12G4); corresponding to antennal S-chaetae type h *sensu*Lukić et al. (2015) and Jantarit and Sangsiri (2020).

Type 5–short, thin, bent, hyaline mic (sometimes looks dark) (7–8 µm) (Fig. 12G5); corresponding to antennal S-chaetae type k *sensu*Lukić et al. (2015) and Jantarit and Sangsiri (2020).

Type 6–short, thin, erected and dark mic (6–12 µm) (Fig. 12G6); corresponding to antennal S-chaetae type g *sensu*Lukić et al. (2015) and Jantarit and Sangsiri (2020).

Type 7–rather long, bent, hyaline mic, thinner distally and broad basally (7–12 µm) (Fig. 12G7).

Type 8–rather long, thin, erected and hyaline mic (sometimes looking dark) (10–15 µm) (Fig. 12G8).

Type 9–long, subcylindrical, bent, hyaline mic (12–14 µm) (similar to type 10 but smaller and thinner) (Fig. 12G9).

Type 10–long, subcylindrical, bent, hyaline and rather broad mic (10–15 µm) (Fig. 12G10); corresponding to antennal S-chaetae type l *sensu*Lukić et al. (2015) and Jantarit and Sangsiri (2020).

Type 11–long, thin, erected and dark mic (10–20 µm) (Fig. 12G11); corresponding to antennal S-chaetae type *sensu*Lukić et al. (2015) and Jantarit and Sangsiri (2020).

Type 12–long, thin and hyaline mic (24–30 µm) (Fig. 12G12); possibly corresponding to antennal S-chaetae type e *sensu*Lukić et al. (2015) and Jantarit and Sangsiri (2020).

Type 13– minute, pointed and dark mic (2 µm) (Fig. 7A (arrow), 12G13). This chaetal type was only found on the Ant. IV of two specimens of *T. kae* sp. nov. in which Ant. II and III are fused.

Type 14–rather short, subcylindrical, bent, hyaline (6–8 µm) (Fig. 12G14).

#### Subapical organite of Ant. IV

Short, thick, dark, swollen at tip (4 µm) phanere with protecting chaeta, inserted dorso-internally ca. 20–50 µm from the apex (Fig. 12H). This organite is present in all Entomobryoidea and most Collembola, with limited changes in morphology, size and position.

#### Distribution patterns of antennal phaneres

In this study, ordinary chaetae on a single antenna numbered 483 for *T. meridionalis* sp. nov. and 518 for *T. kae* sp. nov. and were assigned to the 5 categories described above; 208 S-chaetae were numbered for *T. meridionalis* sp. nov. and 207 for *T. kae* sp. nov. and were assigned to the 14 morphological categories described above. (Tables 4, 5). The ventral side of antenna is richer in both S-chaetae and ordinary chaetae than the dorsal side (ordinary chaetae = 278 vs. 240 in *T. kae* sp. nov., and 251 vs. 232 in *T. meridionalis* sp. nov.; S-chaetae = 115 vs. 92 in *T. kae* sp. nov., and 134 vs. 76 in *T. meridionalis* sp. nov.) (Tables 4, 5). The distribution of the different types of S-chaetae along the antennal segments is arranged in more or less clearly defined patterns which are described in the following paragraphs, summarised in Tables 4 and 5, and illustrated in Figures 3–7, 11H–I, 12A–D, 13, 14.

**Table 4.** Detailed distribution of antennal chaetae. Presence in *Verhoeffiella* and *Alloscopus* by comparison with chaetal morphologies described by Lukic et al. (2015, 2018) and Jantarit and Sangsiri (2020) respectively, ? = no information.

Type of chaetae	Distribution on antenna	Location	Position on antennal segment	Number of chaetae		Presence in *Verhoeffiella*	Presence in *Alloscopus*
				*T. meridionalis* sp. nov.	*T. kae* sp. nov.		
Type 1	Ant. III	dorsal	latero-proximal	1	1	×	×
Type 2	Ant. I	ventral	basal	4	4	–	–
	Ant. II	dorsal	basal	2	2	–	–
Type 3	Ant. I	ventral	proximal	2	2	–	–
	Ant. II	ventral	proximal	1	1	–	–
Type 4	Ant. III	dorsal	AIIIO	2	2	×	×
Type 5	Ant. I	ventral	all segment	7	6	×	×
	Ant. III	dorsal	AIIIO	2	2	–	–
Type 6	Ant. I	dorsal	basal	3	3	×	×
Type 7	Ant. II	ventral	all segment	3	7	–	–
	Ant. III	dorsal	lateral	0	2	–	–
	Ant. III	ventral	middle to proximal	3	4	–	–
	Ant. IVa	dorsal	middle to proximal	4	4	–	–
Type 8	Ant. I	ventral	all segment	6	10	–	–
	Ant. II	dorsal	middle to proximal	9	8	–	–
	Ant. II	ventral	all segment	9	9	–	–
	Ant. III	dorsal	all segment	10	15	–	–
	Ant. III	ventral	middle to proximal	11	6	–	–
	Ant. IVa	dorsal	all segment	9	10	–	–
	Ant. IVa	ventral	all segment	13	5	–	–
	Ant. IVb	dorsal	all segment	9	9	–	–
	Ant. IVb	ventral	all segment	11	0	–	–
Type 9	Ant. I	ventral	latero-proximal	2	1	–	–
	Ant. II	ventral	proximal	2	4	–	–
Type 10	Ant. I	ventral	latero-proximal	14	5	×	×
	Ant. II	ventral	proximal	2	5	–	×
	Ant. III	dorsal	upper middle	0	1	–	×
	Ant. III	ventral	proximal	1	4	–	×
	Ant. IVa	dorsal	middle to proximal	1	3	–	×
	Ant. IVb	dorsal	middle	2	3	–	×
Type 11	Ant. IVa	dorsal	latero-proximal	0	1	×	×
	Ant. IVb	dorsal	all segment	19	22	×	×
	Ant. IVb	ventral	all segment	35	36	×	×
Type 12	Ant. I	ventral	all segment	8	6	×	×
Type 13	Ant. IVa	dorsal	middle of Ant. IVa	0	3	–	–
Type 14	Ant. II	dorsal	proximal	1	1	–	–
Subapical organite	Ant. IVb	dorsal	proximal near the tip	1	1	×	×
Ordinary chaetae	Ant. I	dorsal	all segment	13	28	×	×
	Ant. I	ventral	all segment	27	38	×	×
	Ant. II	dorsal	all segment	63	67	×	×
	Ant. II	ventral	all segment	68	77	×	×
	Ant. III	dorsal	all segment	45	38	×	×
	Ant. III	ventral	all segment	36	45	×	×
	Ant. IVa	dorsal	all segment	62	59	×	×
	Ant. IVa	ventral	all segment	53	60	×	×
	Ant. IVb	dorsal	all segment	49	48	×	×
	Ant. IVb	ventral	all segment	67	58	×	×
**Overall**				692	726	?	945

**Table 5.** Number of chaetae of each type along antennal segments.

Type of S-chaetae/antennal segment	*Troglopedetes meridionalis* sp. nov.						*Troglopedetes kae* sp. nov.					
	Ant. I	Ant. II	Ant. III	Ant. IV		Total	Ant. I	Ant. II	Ant. III	Ant. IV		Total
				Ant. IVa	Ant. IVb					Ant. IVa	Ant. IVb	
Type 1	–	–	1	–	–	**1**	–	–	1	–	–	**1**
Type 2	4	2	–	–	–	**6**	4	2	–	–	–	**6**
Type 3	2	1	–	–	–	**3**	2	1	–	–	–	**3**
Type 4	–	–	2	–	–	**2**	–	–	2	–	–	**2**
Type 5	7	–	2	–	–	**9**	6	–	2	–	–	**8**
Type 6	3	–	–	–	–	**3**	3	–	–	–	–	**3**
Type 7	–	3	3	4	–	**10**	–	7	6	4	–	**17**
Type 8	6	18	21	22	20	**87**	10	17	21	15	9	**72**
Type 9	2	2	–	–	–	**4**	1	4	–	–	–	**5**
Type 10	14	2	1	1	2	**20**	5	5	5	3	3	**21**
Type 11	–	–	–	–	54	**54**	–	–	–	1	58	**59**
Type 12	8	–	–	–	–	**8**	6	–	–	–	–	**6**
Type 13	–	–	–	–	–	**0**	–	–	–	3	–	**3**
Type 14	–	1	–	–	–	**1**	–	1	–	–	–	**1**
Overall S-chaetae	46	29	30	27	76	**208**	37	37	37	26	70	**207**
Subapical organite	–	–	–	–	1	**1**	–	–	–	–	1	**1**
Overall ordinary chaetae	40	131	81	115	116	**483**	66	144	83	119	106	**518**
Total	86	160	111	142	193	692	103	181	119	145	177	726

**First antennal segment**: eight types of S-chaetae are recognised (Figs 3A, B, 11H, 12A–D): type 2, type 3, type 5, type 6, type 8, type 9, type 10, and type 12. Only one type is present on the dorsal side (type 6), the others are located on the ventral side.

**Second antennal segment**: seven types of S-chaetae are recognised (Figs 3C, D, 11H, 13D–F): type 2, type 3, type 7, type 8, type 9, type 10, and type 14. Three types are present on the dorsal side (type 2, type 8 and type 14) and five on the ventral side (type 3, type 7, type 8, type 9, and type 10).

**Third antennal segment**: six types of S-chaetae are recognised (Figs 4A–C, 11H, I, 13A–C): type 1, type 4, type 5, type 7 type 8, and type 10. All are present on the dorsal side and three types are present on the ventral side (type 7, type 8, and type 10).

**Fourth antennal segment**: five types of S-chaetae are recognised (Figs 5A, B, 7A, B, 11H, 14A–C): type 7, type 8, type 10, type 11, and type 13. All are present on the dorsal side, but only two types are present on the ventral side (type 8 and type 11). They are distributed as follows on each subsegment.

**Fourth antennal segment I (a)**: five types of S-chaetae are recognised: type 7, type 8, type 10, type 11 and type 13 (*T. meridionalis* sp. nov. has only three types while *T. kae* sp. nov. has all types), all present on the dorsal side.

**Fourth antennal segment II (b)**: three types of S-chaetae are recognised: type 8, type 10, and type 11, all present on the dorsal side. Two types are present on the ventral side (type 8 and type 11).

The most frequent S-chaetae are type 8 and type 10 that are present all along antennal segments, followed by type 7 that was found on three antennal segments (Ant. II, III, IVa), but not on Ant. I. Type 2, type 3, and type 9 were found on only two segments (Ant. I and Ant. II). Type 5 was found on only two segments (Ant. I and Ant. III). Other types are all limited to a single antennal segment: Ant. I (type 6 and type 12), Ant. II (type 14), Ant. III (type 1, type 4) or Ant. IV (type 11, and type 13 in *T. kae* sp. nov.) (see Tables 4, 5 for details).

With regard to the abundance of S-chaetae along antennal segments, type 8 is the most common followed by type 11 (Tables 4, 5). Ant. IV has the highest number of S-chaetae in both species followed by Ant. I, III, and II respectively in *T. meridionalis* sp. nov. and Ant. III, I and II respectively in *T. kae* sp. nov. (Table 5), while ordinary chaetae abundance ranks as Ant. IV, II, III and I (Table 5). The distal subsegment of antenna IV (Ant. IVb) is richer in S-chaetae than the proximal subsegment Ant. IVa, but their respective number of ordinary chaetae is rather similar (Table 5).

## Discussion

The diversity of chaetal types was very similar in the two studied species, and between-species differences in the relative numbers of each chaetal type were limited, probably only reflecting individual variability. Similarities with *Verhoeffiella* and *Alloscopus* have been noted, but there was also many differences regarding the types of chaetae. It is not clear whether chaetal morphologies which seem special to one of the three genera are really taxon-specific receptors, or the result of undetected homologies due to different morphological evolution of some chaetae in the two genera. The only way to test these hypotheses will be to investigate thoroughly Entomobryoidea of other lineages.

The antennal phaneres of *T. meridionalis* sp. nov. and *T. kae* sp. nov. are arranged in a complex pattern. On the 20 morphological types of chaetae that we recognised (five types for ordinary chaetae, 14 types for S-chaetae and subapical organite of Ant. IV), 12 types were located at a fixed position on antennal segments (four types of ordinary mes; S-chaetae type 1, type 4, type 6, type 11, type 12, type 13, and type 14; subapical organite of Ant. IV) (Tables 4, 5). It would be expected that the number of chaetae should directly vary with the length of antennal segments, ranked as Ant. IV > II > III > I. This applies only to the overall result and to ordinary chaetae (Table 5) but not or only partly to S-chaetae. In particular, Ant. IV (a and b) is longer than other segments, but less rich in types of S-chaetae (Tables 4 and 5). Interestingly, however, the distal subsegment of antenna IV (Ant. IVb) possesses more kinds and a higher density of S-and ordinary chaetae than the proximal one (Ant. IVa), with a complex arrangement of type 11 S-chaetae (Figs 5A, B, 7A–B, 11H, 14A–C). Ant. II, though longer than Ant. I or Ant. III, has a similar or lower number of S-chaetae than Ant. I and III, but it bears more ordinary chaetae than the others (Tables 4, 5). Ant. I, the shortest segment, is proportionally the richest in diversity and number of S-chaetae (eight different types, Tables 4, 5), highlighting the importance of this segment for sensory reception. Ant. III, as common in Collembola, bears a complex sensorial structure (AIIIO) with typically five S-chaetae, conserved across most species of Collembola and widely used in taxonomy (Figs 4A–C, 11I). Antennae appears therefore as a mosaic of sensorial areas, with probably different sensorial functions which remain undocumented in Entomobryoidea.

It is rather common that Ant. II and III fuse together (Fig. 6A–C). It can be found asymmetrically in a single antenna or in both antennae, making the antenna(e) a little shorter (Deharveng and Gers 1993; Lukić et al. 2018), with a chaetotaxic pattern strongly modified. For example, in *T. kae* sp. nov., S-chaetae type 13 is found only in the specimens with fused Ant. II and III. Such fusing may be due to regeneration after the loss of antennal segments following predator attack as observed by Ernsting and Fokkema (1983) in *Orchesella*.

S-chaetae on antennal segments vary in number and probably type diversity, depending on size, age, and sex of Collembola, but this remains to be documented. They are also related to species ecology. Cave adapted species in particular are said to have more developed sensory structures than surface species (Deharveng 1988b; Thibaud and Deharveng 1994; Lukić 2019). A group of subcylindrical S-chaetae (type 10) on antennal segments II and III is for instance present in three Mediterranean cave species of the genus (*T. ruffoi* in Delamare-Deboutteville 1951 and Fanciulli et al. 2003, and *T. absoloni* and *T. ildumensis* in Soto-Adames et al. 2014). This character, however, is unknown in other Mediterranean species. It is absent in the described Thai species, all of which are so far cave-restricted, where chaetae of type 10 never clusters, and are in significantly lower number (Figs 3C, D, 4A, B, 13A–F). The multiplication of type 10 S-chaetae cannot be therefore considered a troglomorphic character at the moment. This character has evolved in caves for Mediterranean lineages cannot be ruled out, but this could only be confirmed by examination of antennal morphology of surface species in the region, which has not been done so far. Nevertheless, antennal elongation observed in most cave *Troglopedetes* is associated to an increase of the number of antennal receptors.

Antennal chaetotaxic characters are widely used for the supraspecific taxonomy of Poduromorpha (Deharveng 1981), but much less in Entomobryoidea (Chen and Christiansen 1993; Deharveng and Bedos 1996; Lukić et al. 2015, 2018), due to their complexity. The diversity and pattern of antennal chaetae described in this work are intended serve as reference for further comparisons in this respect. The recognised chaetal types need to be homologised in morphology and distribution across more genera of Entomobryoidea, and limited data available in the literature or from personal observation already indicate that this is possible in many cases. Knowledge of antennal S-chaetae in Entomobryoidea may prove to be as taxonomically significant as it has been in Poduromorpha.

In a broader context, the complexity of chaetal types and distribution patterns described illustrate the functional complexity of arthropod antennae. Though information is lacking, chaetal pattern of distribution on antennae is probably mostly related to their function and the abiotic environment (light, soil, water, food) and interaction within communities. Antennal sensilla ultrastructure and functions in Collembola were studied by Altner’ team in several papers (e.g., Altner and Kuhn 1989), but very few taxa and antennal sensilla have been examined, e.g., Waldorf (1976), Verhoef et al. (1977), Altner and Thies (1978), Slifer and Sekhon (1978). In a further step, the chaetal types we recognised here, useful for taxonomical purpose, will have to be assigned as far as possible to the categories used by morphologists (Zacharuk 1985), in order to gain insight into their functional organisation on the antenna.

## Supplementary Material

XML Treatment for Troglopedetes

XML Treatment for Troglopedetes kae

XML Treatment for Troglopedetes meridionalis
